# Psychometric Model for Service Firm's Intellectual Stress Diagnosis and Management: Development and Validation

**DOI:** 10.1155/tswj/1117495

**Published:** 2025-05-24

**Authors:** Andy Erhinyoja Emuobonuvie, Godspower Osaretin Ekuobase

**Affiliations:** ^1^Department of Computer Science, University of Benin, Benin City, Nigeria; ^2^Department of Computer Science, University of Delta, Agbor, Nigeria; ^3^Service Science Laboratory, Department of Computer Science, University of Benin, Benin City, Nigeria; ^4^SPESSE UNIBEN, University of Benin, Benin City, Nigeria

**Keywords:** intellectual capital, intellectual stress, job satisfaction, knowledge management, PLS-SEM, service firm, stress management, TTSC

## Abstract

A modern service firm—a complex interaction of humans, technology, and corporate culture driven by knowledge and digitalization—is susceptible to intellectual stress (istress) as do humans. This is because both have intellectual capability—intellectual capital (IC) for service firms and competence for humans. However, studies on the istress of firms are famished. As with humans, addressing this issue will help improve the value production capability and competitiveness of service firms. This study sets for itself two objectives: (i) identify the intellectual stressors (istressors) and inhibitors of istress on service firms and (ii) develop and validate a model for the diagnosis and management of istress in service firms. Mediating the transactional theory of stress and coping (TTSC) model, a conceptual model was formulated. The candidate constructs and indicators of the model were extracted from the literature via systematic and scoping reviews. These constructs were aggregated and streamlined into a questionnaire consisting of 108 indicator questions. The questionnaire was randomly administered online using Google Forms. A total of 185 (55.89%) valid individual responses were codified and loaded into SmartPLS4 for PLS-SEM assessments of the conceptual model including its ablated forms. The standard istressors and inhibitors of istress on firms have been validly identified. The istress value for the service industry has been estimated as −0.323. An operational model for service firm's istress diagnosis and management, christened MISS, has been developed and validated. MISS is capable of reverting istress of service firms from distress to eustress. The MISS implementation guide is also presented.

## 1. Introduction

Stress is an individual's psychological, physiological, and behavioral reaction to stimuli called stressor [1–4]. Stress is a common health phenomenon, particularly in the worklife of an individual, that stealthily impacts his overall health (i.e., the physical, mental, and social well-being) and performance [5–7]. The term stress is often misconstrued to be negative (distress) whereas stress could be positive (eustress) to human health and performance [8–11]. Stress could therefore result in increased or decreased value production capability of a person compelled by stressor(s).

Work and the workplace remain the notable stressors of humans [12–14]. Existing literature on stress, including technology-induced stress (technostress), has been about the natural person [15–18] without recourse to the juristic person (i.e., the workplace)—a complex interaction of humans, technology, and corporate culture—which can also be stressed. However, unlike with humans, the physiological burden of technostress on service firms is void—leaving them with only the cognitive and behavioral burdens. Technostress devoid of physiological burden is what is termed intellectual stress (istress) in this study. This isolation is necessary to enable the understanding and management of technostress on intelligent systems—like smart factories and service systems—that can autonomously perform organizational tasks or jobs.

The modern service firm is a service system—a value coproduction workplace [19, 20] driven by knowledge and digitalization [21]. Knowledge and digitalization make intellectual capital (IC) innate to modern service firms [22–25]. The IC of a firm is an intangible asset of the firm—a core issue in organizational psychology [26, 27]—which consists of its collective knowledge and other resources that delineate the firm's value production capability [28–30]. A firm's IC is akin to human competence—the value production capability of the natural person [31]. Competence, therefore, is to humans what IC is to service firms—their intellectual capability. The intellectual capability of a person can be eroded or improved over time depending on possible stimuli encountered and the mitigation mechanism in place [32]. Undeniably, therefore, since modern service firms possess intellectual capability, they are also capable of suffering from istress amidst intellectual stressors. It is therefore evident that a workplace, particularly a modern service firm, can be stressed intellectually.

istress is, obviously, an aspect of cognitive stress—a reactionary state of entities with intellectual capability [33–36]. The receptor of istress stimuli is the entity's intellectual organ or component. However, irrespective of the istress receptor organ or component of an entity, like every other stress, the overall wellbeing of the intellectually strained entity as a whole is impacted [37, 38]. istress, like its parent technostress, may have similar stressors (e.g., cognitive overload and techocomplexity) which are job- and technology-oriented, and nonphysiological outcomes such as service efficiency, service quality, and customer satisfaction. These notwithstanding, some physiological or psychological stress outcomes (e.g., pain, fatigue, burnout, and turn away) will manifest differently as reduced efficiency, accuracy, reliability, and responsiveness, and even as nonresponsiveness by an intellectually strained entity, unlike with technostress on humans, in the workplace.

Though studies on human cognitive stress abound [33–36], istress of firms appears a blind spot to organizational psychologists, service scientists, and managers of service firms. This blind spot is the focus of this study. To illuminate the concept of istress in the service industry and guarantee its effective management for improved value production and competitiveness in the industry, the study has set for itself the following two objectives: (i) identify the istressors and istress inhibitors of service firms and (ii) develop and validate a psychometric model for the diagnosis and management of istress in service firms.

### 1.1. Conceptual Framework and Hypothesis Development

Industrial and organizational (I/O) psychologists have established foundational organizational stress theories and models that have been adopted or adapted to understand and manage work-related stress in the workplace for improved effectiveness and well-being of both the employee and the organization [39]. However, these theories and models are hinged on the three manifestations of job stress (strain and response): cognitive, psychological, and physiological [39–45]—making them unsuitable for nonbiological intellectually strainable “workers” in modern technology-driven organizations such as the modern service firms.

The foundational transactional theory of stress and coping (TTSC) [45] was adapted for this study because istress, by default, is technology-induced, compelling the assumption of resource availability in the workplace. This assumption of resource (technology) availability associated with technology-induced stress in technology-driven organisations like service firms eliminates the consideration of the job demand resource theory [42] which has a contrary assumption of possible nonavailability of resources. Other foundational theories of stress are as follows: the person–environment (P-E) fit theory [40], the person–technology (P-T) fit theory [46], job demand–control (JDC) theory [44], conservation of resource (COR) model [43], Cooper and Marshall's model [41], and the allostatic load model [39]—which are more physiological and (natural) person-oriented, and hence, inappropriate for the study. However, the TTSC model made evident that stress, its control or mitigation, in an entity emanates from interactions with other available entities [47–50]. The TTSC model is simplistic and generic due to its conceptual abstraction of stress as the interaction between the stressor and the strained variable, thereby permitting the escape of stress from its physiological enclosure. This enables the study and management of job stress on nonbiological intelligent systems.

A modern service firm is driven by specialized task(s), knowledge, digital technology, human competence and wellbeing, and corporate culture or policies [51–56]. These everchanging drivers collectively and continuously mould or strain the organizational competence or IC [57]. The implication of this, in the strict sense of the age-long TTSC, is that the cause and control of istress in service firms reside with these drivers. While knowledge, human competence and wellbeing, and corporate culture are corporate constituents of a firm's IC, technology and tasks are external to a firm's IC [58, 59]. The corporate components of a firm's IC can be reduced to knowledge management (KM) and job satisfaction (JS) in the firm, for simplicity. KM consists of knowledge acquisition (KA), knowledge distribution (KD), and knowledge application (KAP) [60, 61]. The JS of employees delineates their overall wellbeing in the workplace [62]. Technology is the instrument used by firms to perform task(s) [58, 59]. Invariably, therefore, istress in firms occurs when a task(s) and the technology employed place intellectual demands on the firm exceeding its IC. Task and technology are therefore the possible sources of istressors and istress inhibitors in firms while KM and JS are possible mediators—for they typically influence the value production capability of firms. Literature on the relationship among IC, JS, and KM, in the context of organizational stress, is scarce. The impacts of KM and JS on each other in the workplace are inconclusive [63, 64]. This study also addressed these challenges.

**Figure 1:**
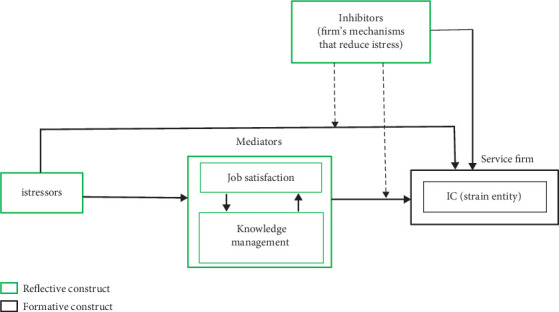
Conceptual model of intellectual stress for service firms.

Integrating the TTSC model [65] and the general mediation model [66], the conceptual model for this study named conceptual model of intellectual stress for service firms (CMISS) is depicted in Figure 1. The CMISS model, unlike existing models of workplace stress that only relate work and other entities of the workplace as stressors with humans as the strained entity, relates the workplace itself as the strained entity. Besides, the CMISS model does not concern itself with the physiological strain on systems that do not do job in the workplace—making it suitable for understanding and managing job stress on service systems.

However, resulting from the controversy surrounding the direction of impact between KM and JS [63, 64] and the acyclic nature of TTSC, the following hypotheses are proposed to help concretize CMISS.

**Hypothesis 1 .**

JS exclusively mediates istress in service firms.

**Hypothesis 2 .**

KM exclusively mediates istress in service firms.

**Hypothesis 3 .**

KM and JS are mutually exclusive mediators of istress in service firms.

**Hypothesis 4 .**

In a mutually inclusive mediation of istress, KM impacts JS.

**Hypothesis 5 .**

In a mutually inclusive mediation of istress, JS impacts KM.

As shown in Figure 1, it is the service firm that reacts despite the firm's IC being the strain variable resulting in possible firm relapse, inefficient service delivery, poor return on investment, disaffection among staff, customers, and suppliers, and eventual fold-up [67, 68]. This is consistent with human stress where cognitive stress can put the entire being in a flight or fight mode [69]. Also, shown in Figure 1 is that the service firm's istress coping mechanism is capable of moderating the mediator's impact on the firm's IC and, as well, directly impacting the IC of the firm. The moderating lines to the paths entering the IC are intended to mitigate the istressors effect on IC, as the issue under study is the istress of a service firm's IC triggered by the istressors.

## 2. Materials and Methods

**Figure 2:**
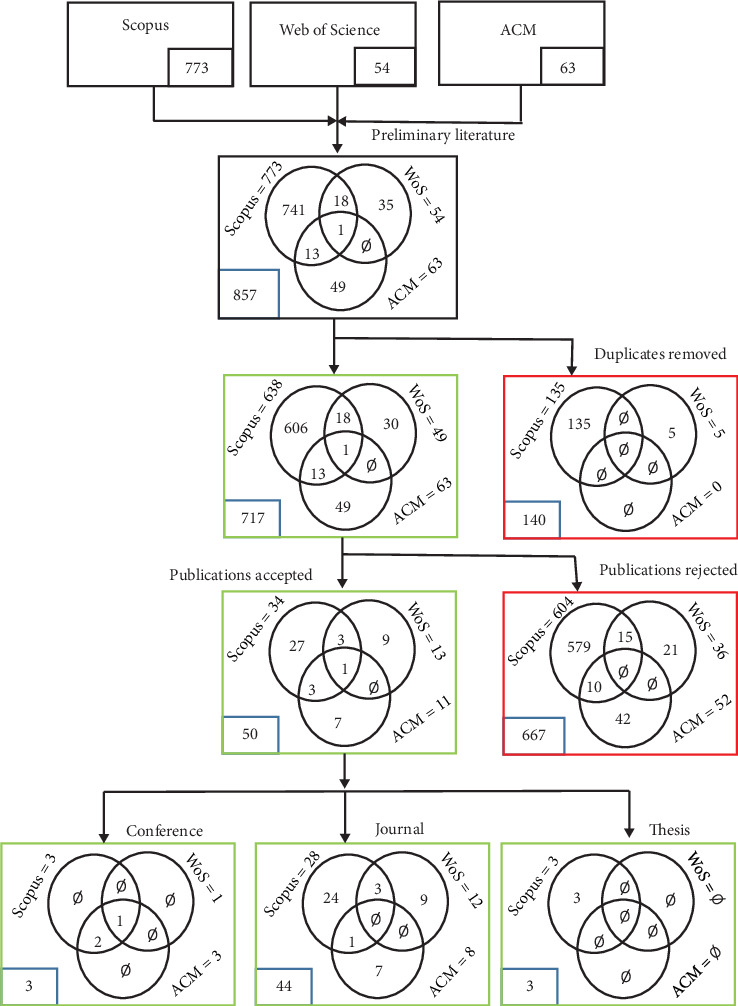
The systematic literature review stages.

The first phase of this study identified the possible istressors and inhibitors of istress in a service firm. This phase was realized via a literature survey, a systematic literature review in particular. Three popular and reputable electronic databases were relied on for the literature review: (i) association of computing machinery (ACM), (ii) Elsevier Scopus (Scopus), and (iii) Web of Science (WoS). Keywords related to the key variables and concepts within the intent and scope of the study were carefully crafted and converted into a search string using the “AND”, “OR”, “(“ and “)” operators to realize the single search item namely, ((“Technostress” OR “Intellectual stress” OR “Intellectual fatique” OR “IT firm”) AND (“Human capital” OR “Knowledge” OR “Tacit knowledge” OR “Skill” OR “Talent” OR “Experience” OR “Training” OR “Competence” OR “Intelligence”)). The search results were examined on a prima facie depth using their titles, abstracts, and keywords resulting in the preliminary literature (*N* = 857) for the study as shown in Figure 2. Thereafter, duplicate pieces of literature were removed. The remaining literature (*N* = 717) was then subjected to inclusion and exclusion criteria as shown in Table 1 resulting in 50 literature.

The accepted literature (*N* = 50), as shown in Figure 2, was then perused for possible istressor and inhibitor constructs extraction by the authors. A total of six istressor and three inhibitor constructs and their scales were finally extracted after the identified possible istressor and inhibitor construct scales underwent construct conciliation and filtering by the authors to eliminate redundancy, imprecision, semantic multiplicity, and vagueness. Furthermore, a scoping review [70, 71] of literature was carried out on literatures retrieved from the ACM, Scopus, and WoS databases, from 15^th^ to 21^st^ March 2022, to identify the scales for each of the mediators and IC constructs in the conceptual model, outside istressors and inhibitors, using each of the construct (or its components) as the search item. The choice of the extracted mediator and IC construct scales selected [72–77] were based on comprehensiveness, universality, simplicity, specificity, and (quantitative) psychometric nature. However, for JS, the generic JS scale [78] was adopted because at the time of the review, no JS scale specifically validated for the technology-driven firms in the global context existed, to the best of our knowledge.

Only the istressor, inhibitor, and JS constructs and their respective indicators already validated in previous technostress studies from the systematic review of literature (*N* = 50) were selected for this study, since istress is a type of technostress. A similar validity criterion was also employed to select the KM and IC constructs and indicators from literatures in the scoping review. The scoping review was done, specifically for the KM and IC constructs because their validated constructs and indicators were not present in the 50 literatures that survived the systematic review. Prominent studies that established the validity of these constructs and indicators are indicated against the constructs as shown in Appendix A.

The selected scales for each of the study variables or constructs were adapted and integrated for the study (see Appendix A) in a 5-point Likert scale of *strongly disagree* (*SD*) (1) to *strongly agree* (*SA*) (5) and merged with the respondent's demographic questions into a single questionnaire, as shown in the Supporting Information section (available here). The questionnaire which consists of two sections—the demographic and construct sections—had a total of 108 indicator questions. The questionnaire was vetted and approved for distribution by the research advisor (second author). The first distribution of the questionnaire was in a pretest study which tested for language clarity, flow of constructs and indicator questions, and the estimated response time needed to respond to the questionnaire. The pretest study was administered online via Google Forms to the then members and associates (*N* = 16) of the Service Science Laboratory (SSL) who were tasked to time themselves. These members and associates of SSL are all information technology (IT) professionals. The use of online in the pretest study as the main study was to get the true feel of the reality.

Some observations from the pretest study resulted in rewording or repositioning the indicator questions under their various constructs and the presentation of the meaning of the constructs in the questionnaire to guide the respondents. The least response time reported was 14 min, and the longest was 18 min. The pretest respondents also complained about the large number of indicator questions. Careful that a large number of indicator questions may result in method bias due to loss of concentration while responding to the questionnaire, the use of procedural remedies akin to those of Ehigbochie and Ekuobase [62] as advocated by Podsakoff et al. [79, 80] were employed in the study to avoid possible common method bias (CMB). The additional introduction of interlude pages in the survey Google Forms after the pretest study resulted in a response time estimate of 15–20 min as indicated in the questionnaire in the Supporting Information section. Thereafter, the questionnaire was randomly administered between 1^st^ April and 30^th^ June 2022 inclusive via the Google Forms. A total of 331 respondents freely consented and participated in the online survey. The respondent's privacy was protected as no respondent's email, Internet protocol (IP) address, or information that can reveal their exact identity was solicited or covertly extracted.

However, only 185 (55.89%) valid respondent's responses, as captured in the Google Form worksheet, were used after screening and cleaning to eliminate incomplete, biased, and nontech workers' responses. The valid respondent's responses to the questionnaire (Section B) were encoded with their numeric equivalent of 1–5 for *SD* to *SA*. Thereafter, the encoded data was uploaded into SmartPLS4 for partial least square structural equation modelling (PLS-SEM). The choice of PLS-SEM is due to the complex nature of the conceptual model of this study and also the formative nature of IC in the conceptual model [81–85]. SmartPLS4 is a notable tool for PLS-SEM analysis [83]. A data size of 185 though below the popular threshold of 200 for PLS-SEM analysis [66] is however acceptable as it exceeds the minimum size of 160 recommended by Memon et al. [66]. Besides, the statistical power analysis conducted using G∗Power (3.1.9.7) indicated 143 as the minimum data size allowable for this study's PLS-SEM analysis with a recommended power (1 − *β*) of 0.80 and effect size (*f*^2^) of 0.15 [66, 86–88].

With the data in SmartPLS4, CMISS was visualized in the SmartPLS4 canvas in its three forms necessitated by the impact direction between the two mediators, if any. Considering CMISS in its three possible forms absolves this study from the controversy surrounding the impact direction between KM and JS [63]. Moreover, this will help establish the truism or otherwise of the hypotheses.

In addition to the procedural remedies employed in this study, the CMB was also assessed through the variance inflation factor (VIF) values of the inner model. As evident in Table K1, the VIF values resulting from the full collinearity test are lower than 3.33. Therefore, these CMISS forms can be considered free of CMB [89].

Thereafter, the PLS algorithm was run for the assessment of the measurement models at the lower order for each of the forms. The resulting factor loadings, composite reliability, Cronbach alpha, average variance extracted (AVE), and heterotrait–monotrait (HTMT) ratio assessment results, as shown in Appendices B, C, and D, satisfied the minimum threshold required to transition to the validation of the higher order constructs [83]. To validate the higher order constructs for each of the forms, the uploaded data updated with the latent variable scores obtained from the PLS algorithm were re-exposed to the PLS algorithm. The resulting factor loadings, composite reliability, Cronbach alpha, AVE, and HTMT ratio assessment results from the second data exposure to the PLS algorithm, as shown in Appendices E, F, and G, satisfied the minimum threshold required to validate the higher order reflective constructs [83]. Also, for the validation of the formative higher order construct (i.e., IC), each of the form's updated data was further exposed to the PLS algorithm resulting in the VIF values and also to bootstrapping resulting in the outer weight statistical relevance and outer loadings values, as shown in Appendix H. The results obtained satisfied the minimum threshold required for formative construct validation [83, 88, 90].

The successful validation of both the lower order and higher order constructs transitioned the model assessment from that of the measurement model to the structural model. The resultant three forms of CMISS with validated constructs were each subjected to the PLS algorithm for the assessment of the VIF of the inner model of the structures. The VIF results of the inner model ruined the possibilities of multicollinearity [83, 89]. With the absence of multicollinearity, the structures were subjected to bootstrapping to validate the relationship among the higher order constructs (direct and indirect). Thereafter, the moderating lines were introduced from the inhibitors to the structural paths entering the IC (*N* = 3) from the two mediators (KM and JS) and the istressors for each of the structures. The resultant structures were then subjected to bootstrapping to ascertain the moderating effect of the inhibitors on those relationships.

After the successful validation of the three forms of CMISS model, an ablation test [91, 92] was performed on the structural validation of CMISS by systematically decomposing the mediators and moderators of CMISS till the barest level of the transaction theory of stress model was realized and iteratively subjected each ablated form to bootstrapping. The structural validation of these models was concluded by assessing the models' explanatory powers. Finally, the stepwise bottom-up synthetic analysis [93] was performed on the structural model assessment results (path coefficients and explanatory power) to realize the operational model for service firm's istress diagnosis and management.

## 3. Results

**Figure 3:**
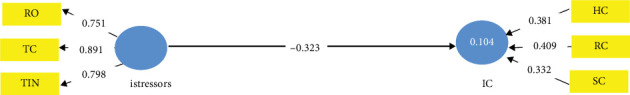
Validated TTS model form of CMISS.

**Figure 4:**
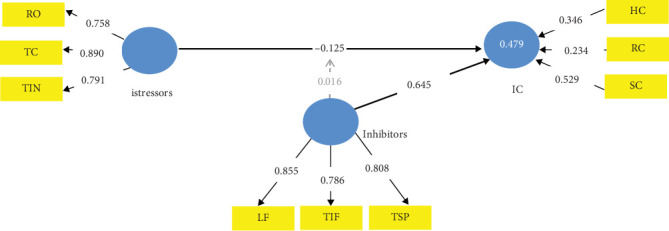
Validated TTSC model form of CMISS.

**Figure 5:**
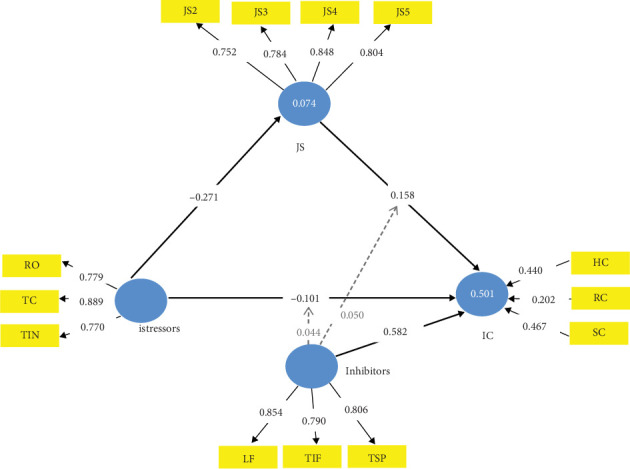
Job satisfaction–mediated TTSC model form of CMISS.

**Figure 6:**
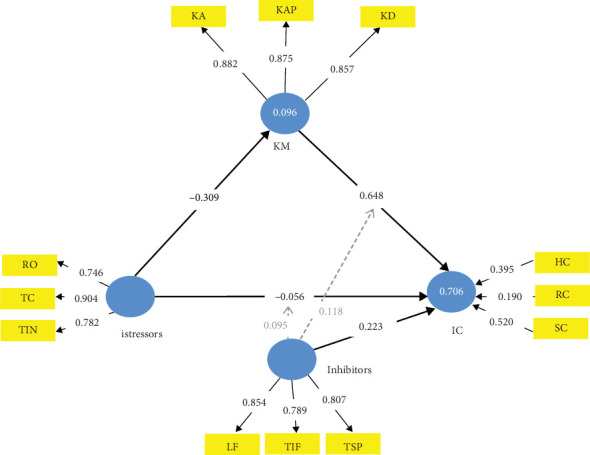
Knowledge management–mediated TTSC model form of CMISS.

**Figure 7:**
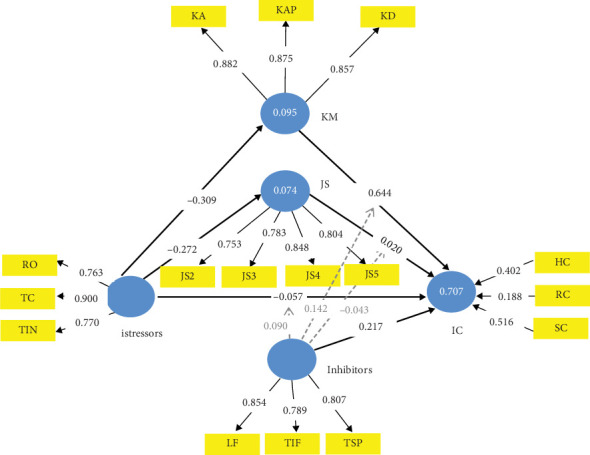
Knowledge management and job satisfaction in parallel mediation of TTSC model form of CMISS.

**Figure 8:**
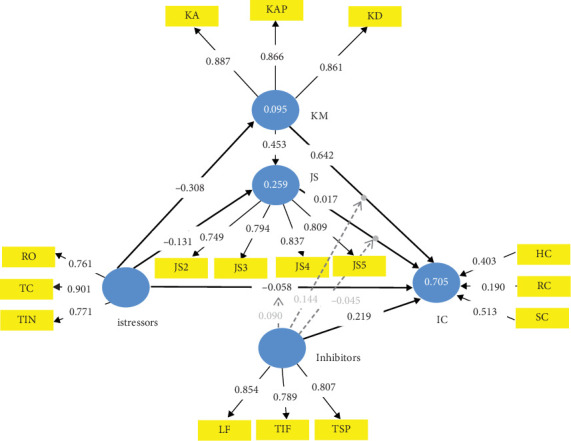
TTSC knowledge management mediating job satisfaction–mediated TTSC model form of CMISS.

**Figure 9:**
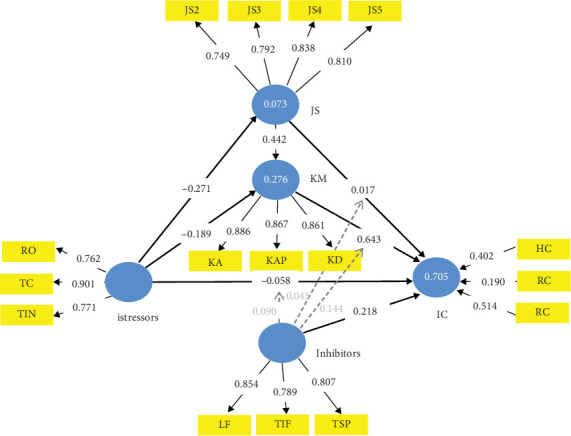
TTSC job satisfaction mediating knowledge management–mediated TTSC model form of CMISS.

Figure 2 captures the details of the systematic literature review. While Appendix A holds the initial CMISS constructs items as identified and integrated from the literature, the Supporting Information section holds the research questionnaire.  Table 2 captures the demography of valid respondents from the online survey. The codified valid respondent's responses (study data) uploaded into SmartPLS4 will be made available upon reasonable request. Appendices B, C, D, E, F, and G hold the measurement model assessment results. The VIF results of the inner model for the three structural forms of CMISS are captured in Table J1 (see Appendix J). Figures 3, 4, 5, 6, 7, 8, and 9 hold the structural forms of CMISS including the ablated forms in increasing order of sophistication. The results of the (moderated) path coefficients, the specific indirect path coefficients, the total effect, and the explanatory power for the structural forms of CMISS including the ablated forms are captured in Tables J2, J3, J4, J5, J6, J7, and J8 (in Appendix J), respectively.  Figure 9 holds the operational model for a service firm's istress diagnosis and management christened MISS. The psychometric scale for MISS implementation is presented in Appendix I.

## 4. Results Interpretation and Implications

As evident in Figure 2, the number of publications (*N* = 50) that were perused for the candidate JS, istressors, and inhibitors scale selection account for 6.97% of the initial publications, excluding duplicates, used for the systematic literature review. This signals that though the literature on stress and its psychometric scales abound only a handful are technology- and knowledge-oriented. The excellent reputation of the literature databases used for both the systematic and scoping literature reviews—WoS, Scopus, and ACM—is a reliable surety for the candidate constructs of this study.

As evident in Table 2, though 55.89% of the total respondents' responses were accepted for this study, 97.37% of the respondents who were IT staff had their responses accepted for the study. This is an indication that the accepted study respondents are in tune with technology-oriented tasks—the predominant tasks of modern service firms. Then, 96.76% of these IT staff are young adults and adults in their prime working age, an indication that the respondents are digitally active personnel. 93.85% of this vibrant class of IT staff are virtually digital natives [94, 95] indicating that the respondents are naturally tech savvy and, thus, adept at technology-oriented psychological issues. At least three of every four of these tech savvy individuals have above high school education indicating an excellent sense of judgement in respondents' responses. Two of every three of the respondents were male (64.86%) and the others were female (34.05%)—an excellent reflection of the global gender spread of modern service firms' employees [96, 97]. The ratio of accepted respondents at the operational, middle, and senior management levels is 6:3:1—an excellent reflection of the proportionate ratio of staff in modern service firms [98]. The breakdown of the workplace of our respondents indicate a good spread within the modern service industry. The captured biographic data of valid respondents signal a comprehensive spread of respondents within the modern service industry, excellent comprehension of the questionnaire, and reliable practical responses. The resultant study data (*N* = 185) from these respondents exceeded in quantity the minimum G∗Power estimate of 143 and the 160 threshold suggested by Memon et al. [66] from their practical experiences. The study data uploaded to SmartPLS4 is therefore sufficient, comprehensive, and reliable.

The measurement model assessment results, as shown in Appendices B, C, D, E, F, and G, all gave a green light to the three forms of CMISS in both the lower order and higher order constructs to transition into the structural model assessment stage. For the measurement model assessment, the green light given was incident on the standard threshold namely, (i) factor loading ≥ 0.7 [83, 99]; (ii) Cronbach alpha ≥ 0.7 [83, 99]; (iii) composite reliability ≥ 0.7 [83]; (iv) AVE ≥ 0.5 [83]; (v) HTMT ≤ 0.85 ≈ 0.9 [83, 99, 100]; (vi) VIF ≤ 3.3 [83, 88, 89]; (vii) sig (outer weight) *p* < 0.05 [83, 90]; and (viii) sig (outer loading) *p* < 0.05 [83, 90]. A significant level of 0.05 was set for the studies because it is sociotechnical research [101–103]. The 100% green light from the measurement model assessments indicates that each CMISS construct with their surviving indicators is well-specified, well-fitted to the study data, and well-aligned.

For the structural model assessment results, the VIF results for the various structural forms of CMISS including the ablated forms are as shown in Table J1. None of the VIF results in Table J1 exceeded the 3.33 threshold [89] indicating that each form of CMISS including the ablated forms accurately represents the underlining relationships between available constructs. Adopting the stepwise bottom-up synthetic approach, the comparative analysis of the path and moderated path coefficients for the structural forms of CMISS including the ablated forms vis-à-vis their associated explanatory power results began with the barest form of CMISS (Figure 3) towards its mediated mediation forms (Figures 7, 8, and 9).

### 4.1. The Barest Forms of CMISS

Figure 3 holds the basic transactional theory of stress (TTS) model without the coping mechanism. From Figure 3, it is evident that the istressors of service firms are (i) role overload (RO), (ii) technocomplexity (TC), and (iii) technoinsecurity (TIN). Their surviving indicators are shown in Appendix I. These istressors are the only three out of the six selected istressors from the literature that survived the SEM framework. The structural assessment result of this CMISS form as shown in Table J2 made evident that istress exists in service firms and puts the istress value at −0.323 with 100.0% (*p* ≤ 0.001) confidence signalling that the service industry is presently in distress intellectually. The implication is that a service firm with proximate istress value may not compete favourably [104, 105]. It is therefore suggested that managers of service firms should regularly monitor the istress level of their firms and strive to keep it sufficiently better than this istress level for the firm's competitiveness. Also shown in Appendix I are the surviving indicators of the three components of IC (human capital (HC), structural capital (SC), and relational capital (RC)) which reduced from 36 to 22 in the complete model. The implication is that only 22 of the 36 conventional IC indicators can expose the presence of istress. Managers of service firms should therefore be on the lookout for these 22 indicator responses in the isolative measurement of their firm's IC as a cue to monitor the istress of their firms. Although this form of CMISS has no moderators and mediators, moderators and mediators are age-long mechanisms for stress management [106].

Figure 4 holds the basic TTSC mechanism model. As evident from the literature, the istress moderators as shown in Appendix I are literacy facilitation, technical support provision, and technology involvement facilitation. Their moderating effect on the firm's istress is evident in the 61.3% reduction of the istress (from −0.323 to −0.125) with 96.5% confidence as evident in Table J3. Also evident in Table J3 is that in this validated CMISS form (TTSC model equivalent), the inhibitors strengthen the firm's IC by 0.645 per its unit improvement with 100% confidence. However, these inhibitors have no significant interaction effect on the istressors (*p* value = 0.394) to influence istress. The istress reduction power of the TTSC model is 0.479 which is moderate by Chin [107] classification. Also, worthy of note is the fact that the effect of the inhibitors (*f*^2^) on istress in this validated CMISS form is 0.723 which is very strong by Cohen [86] classification. The relevance of this information even in the future is *Q*^2^ = 0.439 is also very strong to Hair et al. [108] classification. Managers of start-ups and micro- and small-scale service firms, usually without the requisite funds and expertise for robust KM, can adopt the fundamental TTSC model by leveraging on the inhibitors in the management of istress; as they usually lack formal KM systems [109]. However, irrespective of the scale of a service firm, JS remains a critical psychological issue in the management of stress [110, 111].

### 4.2. TTSC JS-Mediated CMISS Form

Figure 5 holds the TTSC model mediated with JS only. The mediating effect of JS is evident in Table J4. From Table J4, it is evident that the inhibitors with JS collectively strengthen the firm's IC by 0.740 per unit of their improvement, which exceeds the 0.645 from the TTSC model by a factor of 0.095 (i.e., about 15% increase). While the inhibitors contributed 0.582 (78.65%) of this value with 100% confidence, the mediating JS made a significant contribution of 0.158 (21.35%) with 98.5% confidence. The exclusive mediating effect of JS on a firm's istress is evident in the 86.69% reduction of the istress (from −0.323 to −0.043) with 97.3% confidence at its best (specific indirect path) down to 55.42% in total effect (from −0.323 to −0.144) with 97.9% confidence at its worst (direct istressors -> IC path) as evident in Table J3. The implication of this is that JS is an exclusive mediator of istress because its presence as a mediator in the complete TTSC models is inviolable—affirming Hypothesis 1.

Also evident from Table J4 is that inhibitors have no significant influence on JS and the istressors' impact on a firm's IC. The istress reduction power of the TTSC model mediated by JS is 0.501 which, though moderate by Chin [107] classification as with the TTSC model is higher than that of the unmediated TTSC by 0.022 (4.59% increase). Also worthy of note is the fact that the inhibitor's effect on istress in this JS-mediated CMISS form is 0.520 which is very strong by Cohen [86] classification and is therefore critical for a JS-mediated TTSC model. The mediating effect of JS on istress is *f*^2^ = 0.039 which is, however, weak by the same Cohen [86] classification. The relevance of this information in the future is *Q*^2^ = 0.438 which is very strong by Hair et al. [108] classification.

These results imply that managers of start-ups and micro- and small-scale service firms usually devoid of standard KM systems can exclusively, but not immediately, attenuate istress in their firms by ensuring the excellent JS of their staff while leveraging on the inhibitors in the management of istress. Excellent JS is a key here because istressors negatively impact JS almost twice as much as they impact the firm's IC (−0.271: −0.144 ≈ 2:1). This suggestion is logical for a stress mediator has to be able to absolve the stress partially or wholly to shield the strain entity from the stressor's direct or indirect impact [112, 113]. Observe that the specific indirect path through the mediator (JS) weakens the istressors' negative impact on the firm's IC as JS absolves 0.271 (83.90%) of the −0.323 istress value of the service firm's IC. However, this is not foolproof as the predictive relevance of this istressors' impact on JS in this model is 0.059, which is weak by Hair et al. [108] classification.

### 4.3. TTSC KM-Mediated CMISS Form

Figure 6 holds the TTSC model mediated with KM only. This model is particularly useful for medium-to-large-scale service firms usually with formal KM systems. The mediating effect of KM is evident in Table J5. From Table J5, it is evident that the inhibitors with KM collectively strengthen the firm's IC by 0.871 per unit of their improvement, which exceeds the 0.645 from the TTSC model by a factor of 0.226 (i.e., about 35% increase) and the 0.740 from the JS-mediated TTSC model by a factor of 0.131 (i.e., about 18% increase). While the inhibitors contributed 0.223 (25.60%) of this value with 100% confidence, the mediating KM made a contribution of 0.648 (74.40%) with 100% confidence and a mediating effect of *f*^2^ = 0.723, which is strong by Cohen [86] classification. The implication of this is that KM is a better exclusive mediator than JS in the management of istress of modern service firms, thereby affirming Hypothesis 2.

The mediating total effect of KM on the TTSC model is also evident in its capability to significantly revert the istress level of a service firm from distress to eustress not however without the help of the inhibitors. This reverting effect was only possible with KM as a mediator to the TTSC model (see Tables J2, J3, J4, J5, J6, J7, and J8). The implication of this is that with a sound KM system in place in a service firm, the coping mechanisms, as validated in this study, are capable of improving the firm's value addition notwithstanding the istressors' effect on the firm's IC. These facts are in tandem with the prior understanding that KM impacts a firm's IC [114–117].

### 4.4. TTSC KM and JS Mutually Exclusive–Mediated CMISS Form

To complete the discussions on the ablated validated forms of CMISS towards realizing an operational istress model for the service industry, we consider the TTSC model mediated with both KM and JS in parallel as shown in Figure 7. The result of the mediating effect of KM and JS in parallel to the TTSC model is shown in Table J6. From Table J6, it is evident that KM in parallel mediating role with JS in the TTSC model made the mediating role of JS futile as all paths leaving JS into IC had a *p* value above the 0.05 significant threshold, thus, signalling no significant interaction effect of JS on KM to influence istress. This leaves the remaining results almost as that of the KM-only–mediated TTSC model as in Table J5. A Pearson correlation analysis of Tables J5 and J6 with rows involving JS ignored gave a correlation coefficient of 0.999 indicating that the mediating role of JS in Figure 6 is futile. Outside this fact, therefore, a similar discussion as with the KM-only–mediated TTSC model holds for the TTSC model mediated with both KM and JS in parallel. This affirms Hypothesis 3 indicating that both KM and JS cannot be mediators simultaneously (i.e., independently). In particular, in the presence of KM as a mediator in the TTSC model, the independent role of JS as a mediator is not needed.

### 4.5. TTSC KM Mediating JS-Mediated CMISS Form

Although CMISS is cyclic, the TTSC model and PLS-SEM are acyclic. Thus, we considered the direction impact between the two mediators in CMISS—JS and KM—one after the other. Generally, it has been shown that KM impacts JS [63, 64, 118, 119]. However, JS' impact on KM is still fuzzy [63, 64]. Consequently, we begin the discussion of the nonablated forms of CMISS with the direction impact between the mediators going from KM to JS as shown in Figure 8. Table J7 holds the result of the mediating effect of KM and JS with the direction impact between them from KM to JS in the TTSC model. From Table J7, it is evident that the mediating role of JS in the mediated mediation TTSC model with KM mediating JS renders the mediating role of JS futile as all paths leaving JS into IC had a *p* value above the 0.05 significant threshold, thus signalling no significant interaction effect of JS on KM to influence istress. This leaves the remaining results almost as that of the KM-only–mediated TTSC model as in Table J5. A Pearson correlation analysis of Tables J5 and J7 with rows involving JS ignored gave a correlation coefficient of 0.998 indicating that the mediating role of JS in Figure 8 is futile. Outside this fact, therefore, a similar discussion as with KM-only–mediated TTSC model holds for the TTSC model mediated by both KM and JS with the direction impact between the mediators going from KM to JS as shown in Figure 8. These results nullify Hypothesis 4 indicating that in service firms, KM does not impact JS. Thus, existing knowledge that KM impacts JS [63, 64, 118, 119] in knowledge and technology-driven (service) firms is faulty.

### 4.6. TTSC JS Mediating KM-Mediated CMISS Form

The form of the validated CMISS model left for discussion is the TTSC model with the two mediators and the direction impact between them going from JS to KM as shown in Figure 9. Table J8 holds the result of the mediating effect of KM and JS with the direction impact from JS to KM in the TTSC model, that is, JS mediating KM's mediation in the model. It is evident from Table J8 that JS mediating the mediation of KM in the TTSC model significantly reduced the istress value of service firms by 76.16% (i.e., −0.323 down to −0.077) with 99.90% confidence—a mediated mediation fit that was not significant when KM mediated the mediation of JS (see Table J7). The JS mediating effect on KM mediation is evident in its capacity to further reduce the istress value without its direct involvement by 36.36% (i.e., from −0.121 down to −0.077). Furthermore, all the path coefficients from istressor to IC involving KM show a significant istress reduction with or without JS. However, any path from istressors to IC not involving KM had no significant coefficients. This is particularly interesting as though the presence of both mediators in the TTSC model has close absorption strengths in the total effect of about 0.308 and 0.271 for KM and JS, respectively; their cooperative mediation effect was only significant when JS mediated the mediation of KM. Evidently from Table J8 (istressor -> JS -> KM -> IC); therefore, JS significantly interacts with KM in this model to attenuate istress. The implication is that a service firm with a well-established KM system and satisfied employees handles istress better than when the employees are not satisfied. Invariably, the psychological well-being of employees of service firms impacts the firm's IC, as also evident in the total effect section of Table J8.

Also evident from Table J8 is that with JS mediating the mediation of KM, the istressors' impact on the firm's IC via KM was reduced by 61.5% (i.e., −0.2 down to −0.077) directly and indirectly by 39.5% (i.e., −0.2 down to −0.121). The indication of this is that the TTSC model with JS mediating the mediation of KM is a better istress model than the TTSC model mediated with only KM. However, the mediating effect of KM only on the TTSC model managed to be better in the istressors to IC direct path with or without moderation. The explanatory power analysis of this mediated mediation shows that KM and JS are critical constructs that must not be ignored as mediation partners, as shown in Figure 9 and evident from their effect size value in Table J8. The other explanatory power analysis of the KM mediation only is similar to that of the JS-mediated KM mediation, with a correlation coefficient of 0.972. Thus, similar discussions as with KM-only mediation also apply to this model, only with the proviso that the effect size of KM reduced from 0.723 to 0.656, which is equally strong to Cohen [86] classification. These results affirm Hypothesis 5, indicating that, contrary to the popular position [63, 64, 118, 119], JS impacts KM in knowledge-driven firms.

**Figure 10:**
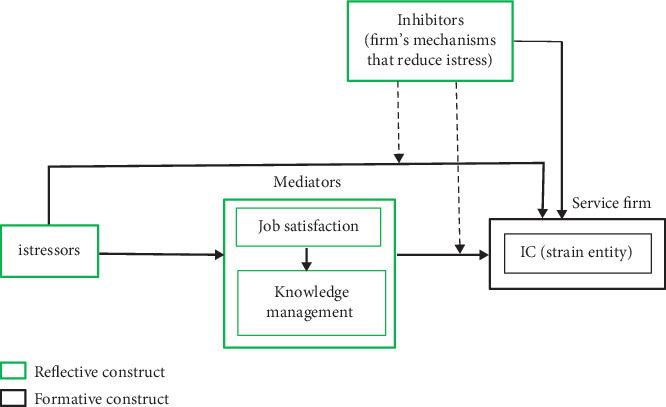
Model of intellectual stress for service firms (MISS).

The overall implication of these discussions is that for start-ups and micro- and small-scale service firms with no KM systems, the JS-mediated TTSC model suffixes. However, for medium- to large-scale service firms usually with established KM systems and the funds and expertise to improve KM, the JS-mediated KM mediation TTSC model is an excellent istress management model. This latter scenario—with JS mediating the mediation of KM on the TTSC model—as shown in Figure 10 has proved to be the most robust istress diagnostic and management model for the service industry. Figure 10 is therefore the operational istress model realized from this study—JS mediating the mediation of KM on the TTSC model christened MISS.

## 5. The MISS Implementation Guide

A standard implementation guide for a psychometric scale is requisite for its seamless and consistent application for enhanced practicality and protection against abuse or misuse [120]. The MISS implementation should begin with the administration of its questionnaire (Appendix I) usually by an experienced I/O psychologist via structured interview or self-assessment report randomly but fairly across the three levels of management (operational, middle, and senior) of each unit and/or branch of the service firm with sufficient sample size. Responses should be screened and codified into their numeric equivalent (from *SD* = 1 to *SA* = 5). Thus, for each construct, we compute their aggregate respondents' ratings, *V*, using Equation (1).

(1) $$\begin{array}{c} V_{t,r,i,j}=\frac{\left(\sum_{r=1}^{n}\left(\sum_{i=1}^{f_{t}}\left(\sum_{j=1}^{k}I_{r,i,j}/5*k\right)/f_{t}\right)\right)}{n}, \end{array}$$

where *V*_*t*,*r*,*i*,*j*_ is the *i*th factor of model variable *V*_*t*_, *t* = 1 (1) *c* is the number of constructs (reflective or formative), *i* = 1(1) *f*_*t*_ is the number of factors in *V*_*t*_ for all *t*, and *j* = 1(1)*k* number of indicators in each factor *V*_*t*,*i*_.

Thereafter, we compute the IN and OUT values for each *V*_*t*_ and significant edge in Figure 9 using Equations (2) and (5). The IN function of a construct defines the aggregate of the significant interaction effect of the impacting construct(s), while the OUT function defines its significant reactionary impact or reaction. From Equation (1), it is evident that the least value of *V* is one. Since, indegree (istressor) = 0, then Equation (2) holds.

(2) $$\begin{array}{c} \text{IN}\left[\text{istressor}\right]=1. \end{array}$$

Otherwise,

(3) $$\begin{array}{c} \text{IN }\left[V_{t}\right]=\sum \text{OUT}\left[\text{pred}\left(V_{t}\right)\right]. \end{array}$$

For all significant edge entry to *V*_*t*_, and

(4) $$\begin{array}{c} \text{OUT}\left[V_{t}\right]=\text{IN}\left[V_{t}\right]*V_{t}*\left(\left(e_{g}\right)\left\{+{\text{in}}_{e}\right\}\right). \end{array}$$

In particular,

(5) $$\begin{array}{c} \text{OUT}\left[\text{IC}\right]=\text{IN}\left[\text{IC}\right]*V_{\text{IC}}. \end{array}$$

For each, if any, significant edge, *e*, leaving *V*_*t*_ and all predecessor constructs with significant edge entry into *V*_*t*_, where *e*_*g*_ is the weight assigned to edge *e*, and in_*e*_ is the reflective weight on edge, *e*, the use of curly braces indicates optionality, that is, used only where applicable. The OUT [IC] approximate the istress level of the firm under investigation. The unique OUT function's definition for IC is predicated on its formative nature, unlike others that are reflective. Equations (1), (2), (3), (4), and (5) are either single or multilevel arithmetic means or multiplier effects of interaction impact whose proof of correctness is trivial. Firms without a standard KM system may use the model as depicted in Figure 5. For classification convenience, the following istress level classification as summarized in Table 3 is suggested.

We further suggest as a simple guide of action, emanating from this study, that when the istress value is in the relaxation stage, managers of service firms may choose to do nothing about the firm's istress. However, at the alert stage, the need for intervention at the level of moderators may become necessary. At the monitoring stage, the JS of the employees can be rejigged while, at the management stage, it is imperative to rejig the KM system if it exists; otherwise, KM practices should be formalized and fully integrated if resources (funds and expertise) are available. However, at the emergency stage, every intervention arsenal of mitigating istress—moderators, enhanced employee satisfaction, and improvement on the KM system must—be engaged. However, where resources for standard KM practices are scarce, the organization may intervene from the area of JS and inhibitors; otherwise, the firm may be exposed to threats of fold up or, at best, an inability to favorably compete. The rejigging should come as tactical and strategic managerial and policy interventions strengthening targeted constructs, as evident from their computed *V*_*t*_ values and inspections of the respective indicator ratings. Subject to the availability of resources, an intellectual stress test (IST) should be performed biannually on a service firm to monitor the istress status of the firm and evaluate the intervention effect.

## 6. Conclusion

The study has shown that istress exists in firms driven by knowledge and technology due to its intellectual capability termed IC. Though the istress of service firms has evaded the attention of organizational psychologists and service scientists, this study has developed and validated a psychometric model, using the PLS-SEM assessments in SmartPLS4, that can help service scientists and managers of service firms ascertain the istress level of service firms—distress or eustress. The conceptual model (CMISS) that birthed the operational model (MISS) was based on the integration of the TTSC model and the general mediation model. The istressors validated by the model are (i) RO with three indicators, (ii) TC with four indicators, and (iii) TIN also with four indicators. Although there were six candidate istressors with 28 indicators in total that underwent PLS-SEM assessment, the other three candidate constructs—techno-overload, technoinvasion, and technouncertainty—and 17 indicators fell out. All the candidate moderator constructs—literacy facilitation, technical support provision, and technology involvement facilitation—and their 13 indicators in total survived as standard firm's IC istress moderator.

For the firm's IC, though all its three formative constructs—HC, RC, and SC—survived only 22 of their 36 candidate indicators survived. The mediators were JS and KM. KM another composite construct consisting of KA, KAP, and KD had all the subconstructs surviving the PLS-SEM assessments. However, only 13 of the 19 candidate indicators survived the PLS assessment. The candidate constructs and their indicators extracted via the systematic and scoping review method were already validated from previous studies. JS, a prime construct, had only one indicator falling out. The data (*N* = 185) fed into the SmartPLS4 for the PLS-SEM assessments—a product of an online questionnaire survey of staff of service firms—are sufficient, comprehensive, and reliable for the study.

From the PLS-SEM assessments, the istress of the service industry has been estimated to be −0.323 at its worst, signaling an intellectually distressed sector without operational KM and moderating interventions. The analysis of the validated CMISS model and its ablated forms made evident that KM is an inviolable mediator for the management of istress of firms, for its presence as a mediator in the TTSC model is capable of reverting istress of firms from distress to eustress. However, for start-ups and micro- and small-scale service firms with limited KM systems, if any, JS and moderators were shown to be good enough to manage the firm's istress.

In all, this study developed and validated a TTSC model, christened MISS, for the diagnosis and management of a special type of technostress termed istress as depicted in Figure 10. The MISS model extends the understanding of organizational stress beyond employees in the workplace to the workplace itself, particularly for knowledge and technology-driven firms such as a modern service firm. The model made evident that the JS of the employees of service firms strengthens the mediation power of KM systems on firms' istress. Thus, contrary to the popular position, it is now evident that JS impacts KM in knowledge-driven firms. Managers of service firms are enjoined to regularly monitor and subdue their firm's istress using the MISS model for enhanced organizational effectiveness. The implementation guide to do this is succinctly presented in this study.

### 6.1. Limitation of Study and Suggestion for Further Studies

A notable limitation of this study is that it was validated only in the context of the English language despite its global respondents from diverse sectors of the service industry. The need to revalidate the operational mediated mediation TTSC model developed and validated in this study in the context of other languages toward universality beckons. The demonstration of the MISS implementation guide, which involves the use of real-life data solicited from selected firms across the small-, medium-, and large-scale service firms based on the MISS questionnaire, will be reported in a different communication to keep this study within its perspective of development and validation. The automation of the MISS implementation guide is encouraged.

## Figures and Tables

**Table 1:** Inclusion and exclusion criteria.

**Inclusion**	**Exclusion**
Publications published between January 2010 and February 2022 inclusive	Publications with contents (partial or whole) written in languages outside the English language
Publication with title, abstract, and keywords in English language only	Publications outside the scope of service firms, and IT industry
Publications within the scope of technostress, intellectual capital, intellectual stress, knowledge management, job satisfaction, organizational stress, and psychometrics	

**Table 2:** Demographics of valid respondents.

**Are you an IT staff**	**Frequency/percentage**	**Gender**	**Frequency/percentage**	**Age**	**Frequency/percentage**	**Education**	**Frequency/percentage**
Yes	190 (57.4%)	Male	120 (64.86%)	Below 20 years	10 (5.40%)	High school	45 (24.32%)
No	141 (42.6%)	Female	63 (34.05%)	20–29 years	111 (60%)	Diploma	28 (15.14%)
		Others	2 (1.08%)	30–39 years	47 (25.41%)	Bachelor ‘s degree	76 (41.08%)
		40–49 years	11 (5.95%)	Master's degree	32 (17.28%)
50–59 years	6 (3.24%)	PhD degree	4 (2.16%)
60 years and above	0 (0.00%)		
**Total**	331 (100%)	**Total**	185 (100%)	**Total**	185 (100%)	**Total**	185 (100%)

**Type of organization**			**Frequency/percentage**	**Level of management**	**Frequency/percentage**		
Automobile, transport & logistics			4 (2.16%)	Operational	114 (61.62%)
Banks finance & insurance			7 (3.78%)	Middle	52 (28.11%)
Consultancy and services			54 (29.19%)	Senior	19 (10.27%)
Education			19 (10.27%)		
Manufacturing, construction, & engineering			8 (4.32%)		
Retail, wholesale, & distribution			6 (3.24%)		
Technology			29 (15.68%)		
Telecommunication			35 (18.91%)		
Others			23 (12.43%)		
**Total**			185 (100%)	**Total**	185 (100%)

**Table 3:** Service firm's distress level classification.

**istress level**	**Classification**	**Remark**
istress < −0.35	Strong IC distress	Emergency stage
−0.35 ≤ istress < −0.15	Moderate IC distress	Management stage
−0.15 ≤ istress < 0.05	Weak IC distress	Monitoring stage
0.05 ≤ istress < 0.15	Weak IC eustress	Alert stage
istress ≥ 0.15	Strong IC eustress	Relaxation stage

**Table 4:** Section B.

**Item number**	**Dear respondent, please indicate your level of agreement or disagreement with the questions below by checking [√] the appropriate option according to the scoring given below.** **[(1) = *strongly disagree*, (2) = *disagree*, (3) = *neutral/undecided*, (4) = *agree*, (5) = *strongly agree*]**		
**Item code**	**Constructs/meaning**	**Questions/items**	**Source**
*Stressors*			
TO1	**Techno-overload:** Depicts situations where IT users/professionals are exposed to more IT that can make them work more and faster than they can conveniently handle	I am forced by this technology to work much faster	[78, 121]
TO2		I am forced by this technology to do more work than I can handle	
TO3		I am forced by this technology to work with very tight time schedule	
TO4		I am forced to change my work habits to adapt to new technologies	
TO5		I have a higher workload because of increased technology	

TI1	**Technoinvasion:** Depicts situations where IT users/professionals can work or be reached anywhere and anytime and feel the need to be constantly connected	I spend less time with my family due to use of technology	[78, 121]
TI2		I have to be in touch with my work even during vacations due to the use of technology	
TI3		I have to sacrifice my vacation and weekend time to keep current on new technologies	
TI4		I feel my personal life is being invaded by use of technology	

TC1	**Technocomplexity:** Depicts situations where IT forces IT professionals/users to spend more time and effort in learning and understanding how to use new applications and technologies	I do not know enough about this technology to handle my job satisfactorily	[78, 121]
TC2		I need a long time to understand and use new technologies	
TC3		I do not find enough time to study and upgrade my technology skills	
TC4		I find new recruits to this organization know more about the technology than I do	
TC5		I often find it too complex to understand and use new technologies	

TIN1	**Technoinsecurity:** Depicts the complex or phobia IT users/professionals have with the use of new technologies	I feel constant threat to my job due to new technologies	[78, 121]
TIN2		I have to constantly update my skills to avoid being replaced	
TIN3		I am threatened by coworkers/peers with newer technology skills	
TIN4		I do not share my knowledge with my coworkers/peers for fear of being replaced	
TIN5		I feel there is less sharing of knowledge among coworkers/peers for fear of being replaced	

TU1	**Technouncertainty:** Depicts situations where the IT skillset required by IT users/professionals is constantly changing and rapidly so	There are always new entrance/development in the technologies used in our organization	[78, 121]
TU2		There are constant changes in computer software in our organization	
TU3		There are constant changes in computer hardware in our organization	
TU4		There is frequent upgrades in computer network in our organization	

RO1	**Role overload:** Depicts situations where IT enables users/professionals to perform multiple roles simultaneously	I perform multiple roles with the help of technology	[122]
RO2		I have to do things that I do not have enough time and energy for	
RO3		I need more hours in the day to do all the things that are expected of me	
RO4		I never seem to catch up	
RO5		There are times when I cannot meet everyone's expectations	

*Stress inhibitors*			
LF1	**Literacy facilitation:** Mechanisms provided by organization management to help IT users or professionals cope with the demands of learning about new technology	Our organization encourages knowledge sharing to help deal with new technology	[78, 121]
LF2		Our organization emphasizes teamwork in dealing with new technology-related problems	
LF3		Our organization provides end-user training before the introduction of new technology	
LF4		Our organization foster a good relationship between IT department and end-users	
LF5		Our organization provides clear documentation to end-user on using new technologies	

TSP1	**Technical support provision:** Mechanisms provided by organization management to address users anxiety about potentially disruptive mistakes and technical problems	Our end-user help desk does a good job of answering questions about technology	[78, 121]
TSP2		Our end-user help desk is well staffed by knowledgeable individuals	
TSP3		Our end-user help desk is easily accessible	
TSP4		Our end-user help desk is responsive to end-user requests	

TIF1	**Technology involvement facilitation:** Mechanisms provided by organization management to keep users informed and familiar with new technology	Our end-users are encouraged to try out new technologies	[78, 121]
TIF2		Our end-users are rewarded for using new technologies	
TIF3		Our end-users are consulted before introduction of new technology	
TIF4		Our end-users are involved in technology change and/or implementation	

*Job satisfaction*			
JS1	**Job satisfaction:** Describes how pleased a worker is with his or her position of employment in the organization or an evaluation of perceived job characteristics, work environment, and emotional experiences	I always exceed my performance expectation at work place	[78]
JS2		I like doing the things I do while working	
JS3		I feel a sense of pride in doing my job	
JS4		My job is enjoyable	
JS5		I am satisfied with the feeling of accomplishment I get from my job	

*Knowledge management behavior*			
KA1	**Knowledge acquisition:** Knowledge acquisition consists of managing and using existing information and capturing new ones.	We have a system that allows us to learn successful practices from other organizations	[74, 76, 77]
KA2		The company is in touch with professionals and expert technicians	
KA3		The organization encourages the employees to join formal or informal networking made up by professionals and experience users from outside the organization	
KA4		We often ask our customers what they want or need	
KA5		The employees attend fairs and exhibitions regularly	
KA6		There is a consolidated and resourceful Research & Development (R&D) policy	
KA7		New ideas and approaches on work performance are experienced continuously	
KA8		The organizational systems and procedures support innovation	

KD1	**Knowledge distribution:** Knowledge distribution refers to managing the sharing of information in an organization, to prompt innovative and creative ideas	All employees are informed about the aims of the company	[76]
KD2		Meetings are periodically held to inform all the employees about the latest innovations in the company	
KD3		The company has formal mechanisms to guarantee the sharing of the best practices among the different fields of the activity	
KD4		Information technology is used to improve the flow of information and to encourage communication between individuals within the company	
KD5		There are individuals within the organization who take part in several teams or divisions and act as links between them	
KD6		There are individuals responsible for collecting, assembling, and distributing internal employees' suggestions	

KAP1	**Knowledge application:** It entails the integration of the knowledge obtained from both the acquisition and distribution phases into daily business processes to enhance the firm's efficiency and effectiveness	Our organization always apply the latest technology in the market/or our organization is always up-to-date in technology application	[72, 75–77]
KAP2		Our employees are well trained in the latest knowledge in their respective position for better job performance	
KAP3		Our training process is relevant and effective to improve performance and productivity	
KAP4		Our organization has processes for applying experimental knowledge	
KAP5		Our organization has processes for applying knowledge to solve new problems	

*Intellectual capital*			
HC1	**Human capital:** The summation of employees' skill, capabilities, experience, education, and attitude towards life and business	Our employees undergo continuous training	[73]
HC2		Our employees are highly educated	
HC3		Our employee skills are always upgraded	
HC4		Our employees are creative and bright	
HC5		Our employees come up with new ideas	
HC6		Our employees are motivated to share new ideas	
HC7		Our employees have innovative ideas	
HC8		Managers in our organization make sure that employees are happy	
HC9		Managers in our organization understands all factors of employee satisfaction	
HC10		Managers in our organization help employees in solving official problems	
HC11		Our employees are generally happy to work	
HC12		Our employees are happy to put extra effort when needed	
HC13		Our employees are devoted to their work	

RC1	**Relational capital:** The output of the organization's relationship with customers, partners, shareholders, and other stakeholders that are critical to the organizational performance	Data about customers are continuously updated in our organization	[73]
RC2		Our organization continuously meet customers	
RC3		Our organization places a great focus on customers' feedback	
RC4		Customers' feedback is shared across departments in our organization	
RC5		Employees enhance their capabilities through interaction	
RC6		Employees solve problems through cooperation	
RC7		Our organization's total customer base is improving	

SC1	**Structural capital:** The mechanism put in place by the organization that helps to support employees for optimum intellectual performance	The atmosphere in our organization is pleasant	[73]
SC2		In our organization, managers and staff communicate well	
SC3		Knowledge increase is well supported in our organization	
SC4		Our organization continuously develops new products and services	
SC5		Our organization support innovative ideas	
SC6		Our organization constantly improves service quality	
SC7		Our organization embeds much of its information in structures and systems	
SC8		Employees have access to the information system whenever needed	
SC9		Our organization possess processes to develop its unique capabilities	
SC10		Our organization culture and atmosphere are supportive and comfortable	
SC11		Our organization uses computers for operational purposes	
SC12		Our organization is embedded with the latest information technology software	
SC13		Information technology contributes to service quality in our organization	
SC14		Our organizational systems and procedures support innovation	
SC15		Employees of our organization are highly empowered	
SC16		Employees of our organization are stimulated to take initiatives	

**Table 5:** Outer/factor loadings.

	**KJ**	**JK**	**Original**
HC11 <- HC	0.739	0.740	0.740
HC12 <- HC	0.747	0.747	0.747
HC6 <- HC	0.781	0.781	0.781
HC7 <- HC	0.728	0.728	0.728
HC8 <- HC	0.792	0.792	0.792
HC9 <- HC	0.805	0.805	0.805
JS2 <- JS	0.749	0.749	0.755
JS3 <- JS	0.798	0.798	0.795
JS4 <- JS	0.835	0.832	0.838
JS5 <- JS	0.807	0.809	0.802
KA3 <- KA	0.721	0.720	0.718
KA6 <- KA	0.710	0.709	0.711
KA7 <- KA	0.823	0.823	0.821
KA8 <- KA	0.828	0.830	0.832
KAP1 <- KAP	0.724	0.727	0.728
KAP2 <- KAP	0.807	0.806	0.805
KAP3 <- KAP	0.817	0.818	0.817
KAP4 <- KAP	0.785	0.784	0.784
KAP5 <- KAP	0.840	0.839	0.840
KD1 <- KD	0.810	0.809	0.810
KD2 <- KD	0.791	0.792	0.789
KD3 <- KD	0.836	0.838	0.836
KD4 <- KD	0.771	0.770	0.774
LF1 <- LF	0.712	0.712	0.712
LF2 <- LF	0.782	0.782	0.782
LF3 <- LF	0.847	0.847	0.847
LF4 <- LF	0.809	0.809	0.809
LF5 <- LF	0.807	0.807	0.807
RC2 <- RC	0.710	0.710	0.710
RC3 <- RC	0.761	0.761	0.761
RC4 <- RC	0.714	0.714	0.714
RC5 <- RC	0.794	0.794	0.794
RC6 <- RC	0.757	0.757	0.757
RC7 <- RC	0.754	0.754	0.754
RO2 <- RO	0.795	0.794	0.795
RO3 <- RO	0.811	0.811	0.811
RO4 <- RO	0.893	0.893	0.893
SC10 <- SC	0.764	0.764	0.764
SC12 <- SC	0.715	0.715	0.715
SC14 <- SC	0.763	0.763	0.763
SC15 <- SC	0.736	0.736	0.736
SC16 <- SC	0.770	0.770	0.770
SC4 <- SC	0.778	0.778	0.778
SC5 <- SC	0.786	0.786	0.786
SC6 <- SC	0.778	0.778	0.778
SC7 <- SC	0.802	0.802	0.802
SC9 <- SC	0.799	0.799	0.799
TC1 <- TC	0.800	0.800	0.800
TC2 <- TC	0.841	0.841	0.841
TC3 <- TC	0.784	0.784	0.784
TC5 <- TC	0.790	0.790	0.790
TI1 <- TI	0.725	0.725	0.725
TI2 <- TI	0.713	0.713	0.713
TI3 <- TI	0.819	0.819	0.819
TI4 <- TI	0.797	0.797	0.797
TIF1 <- TIF	0.679	0.679	0.679
TIF2 <- TIF	0.703	0.703	0.703
TIF3 <- TIF	0.745	0.745	0.745
TIF4 <- TIF	0.743	0.743	0.743
TIN1 <- TIN	0.687	0.687	0.687
TIN3 <- TIN	0.767	0.768	0.767
TIN4 <- TIN	0.722	0.721	0.721
TIN5 <- TIN	0.754	0.754	0.753
TO1 <- TO	0.893	0.893	0.893
TO2 <- TO	0.681	0.681	0.682
TO3 <- TO	0.822	0.822	0.822
TO4 <- TO	0.669	0.670	0.670
TSP1 <- TSP	0.851	0.851	0.851
TSP2 <- TSP	0.769	0.769	0.769
TSP3 <- TSP	0.845	0.845	0.845
TSP4 <- TSP	0.878	0.878	0.878
TU1 <- TU	0.717	0.717	0.717
TU2 <- TU	0.874	0.874	0.874
TU3 <- TU	0.708	0.708	0.707
TU4 <- TU	0.831	0.831	0.831

**Table 6:** Cronbach alpha, composite reliability, and average variance extracted.

	**Cronbach alpha**			**Composite reliability**			**Average variance extracted**		
	**KJ**	**JK**	**Original**	**KJ**	**JK**	**Original**	**KJ**	**JK**	**Original**
HC	0.859	0.859	0.859	0.895	0.895	0.895	0.587	0.587	0.587
JS	0.811	0.811	0.811	0.875	0.875	0.875	0.636	0.636	0.637
KA	0.774	0.774	0.774	0.855	0.855	0.855	0.597	0.597	0.597
KAP	0.854	0.854	0.854	0.896	0.896	0.896	0.633	0.633	0.633
KD	0.816	0.816	0.816	0.878	0.878	0.878	0.644	0.644	0.644
LF	0.852	0.852	0.852	0.894	0.894	0.894	0.629	0.629	0.629
RC	0.843	0.843	0.843	0.885	0.885	0.885	0.561	0.561	0.561
RO	0.799	0.799	0.799	0.872	0.872	0.872	0.696	0.695	0.696
SC	0.923	0.923	0.923	0.936	0.936	0.936	0.592	0.592	0.592
TC	0.821	0.821	0.821	0.880	0.880	0.880	0.647	0.647	0.647
TI	0.762	0.762	0.762	0.849	0.849	0.849	0.585	0.585	0.585
TIF	0.687	0.687	0.687	0.810	0.810	0.810	0.516	0.516	0.516
TIN	0.713	0.713	0.713	0.823	0.823	0.823	0.537	0.537	0.537
TO	0.806	0.806	0.806	0.853	0.853	0.854	0.596	0.596	0.597
TSP	0.857	0.857	0.857	0.903	0.903	0.903	0.700	0.700	0.700
TU	0.798	0.798	0.798	0.865	0.865	0.865	0.617	0.617	0.617

**Table 7:** HTMT ratio.

	**HC**	**JS**	**KA**	**KAP**	**KD**	**LF**	**RC**	**RO**	**SC**	**TC**	**TI**	**TIF**	**TIN**	**TO**	**TSP**	**TU**
*KJ*																
HC																
JS	0.554															
KA	0.775	0.596														
KAP	0.783	0.418	0.799													
KD	0.727	0.542	0.814	0.719												
LF	0.618	0.478	0.735	0.637	0.713											
RC	0.771	0.378	0.628	0.688	0.716	0.662										
RO	0.251	0.258	0.176	0.214	0.107	0.211	0.182									
SC	0.821	0.414	0.753	0.831	0.690	0.662	0.770	0.224								
TC	0.305	0.325	0.398	0.326	0.350	0.318	0.356	0.586	0.325							
TI	0.221	0.152	0.191	0.129	0.085	0.229	0.175	0.710	0.202	0.408						
TIF	0.707	0.399	0.659	0.565	0.560	0.603	0.491	0.157	0.629	0.312	0.160					
TIN	0.264	0.164	0.168	0.175	0.280	0.274	0.281	0.572	0.221	0.756	0.549	0.240				
TO	0.123	0.132	0.117	0.123	0.170	0.115	0.111	0.494	0.117	0.229	0.642	0.159	0.317			
TSP	0.451	0.443	0.601	0.493	0.500	0.667	0.473	0.186	0.529	0.306	0.239	0.613	0.255	0.087		
TU	0.197	0.237	0.361	0.418	0.294	0.364	0.290	0.352	0.317	0.077	0.333	0.186	0.172	0.398	0.220	

*JK*																
HC																
JS	0.554															
KA	0.775	0.596														
KAP	0.783	0.418	0.799													
KD	0.727	0.542	0.814	0.719												
LF	0.618	0.478	0.735	0.637	0.713											
RC	0.771	0.378	0.628	0.688	0.716	0.662										
RO	0.251	0.258	0.176	0.214	0.107	0.211	0.182									
SC	0.821	0.414	0.753	0.831	0.690	0.662	0.770	0.224								
TC	0.305	0.325	0.398	0.326	0.350	0.318	0.356	0.586	0.325							
TI	0.221	0.152	0.191	0.129	0.085	0.229	0.175	0.710	0.202	0.408						
TIF	0.707	0.399	0.659	0.565	0.560	0.603	0.491	0.157	0.629	0.312	0.160					
TIN	0.264	0.164	0.168	0.175	0.280	0.274	0.281	0.572	0.221	0.756	0.549	0.240				
TO	0.123	0.132	0.117	0.123	0.170	0.115	0.111	0.494	0.117	0.229	0.642	0.159	0.317			
TSP	0.451	0.443	0.601	0.493	0.500	0.667	0.473	0.186	0.529	0.306	0.239	0.613	0.255	0.087		
TU	0.197	0.237	0.361	0.418	0.294	0.364	0.290	0.352	0.317	0.077	0.333	0.186	0.172	0.398	0.220	

*Original*																
HC																
JS	0.554															
KA	0.775	0.596														
KAP	0.783	0.418	0.799													
KD	0.727	0.542	0.814	0.719												
LF	0.618	0.478	0.735	0.637	0.713											
RC	0.771	0.378	0.628	0.688	0.716	0.662										
RO	0.251	0.258	0.176	0.214	0.107	0.211	0.182									
SC	0.821	0.414	0.753	0.831	0.690	0.662	0.770	0.224								
TC	0.305	0.325	0.398	0.326	0.350	0.318	0.356	0.586	0.325							
TI	0.221	0.152	0.191	0.129	0.085	0.229	0.175	0.710	0.202	0.408						
TIF	0.707	0.399	0.659	0.565	0.560	0.603	0.491	0.157	0.629	0.312	0.160					
TIN	0.264	0.164	0.168	0.175	0.280	0.274	0.281	0.572	0.221	0.756	0.549	0.240				
TO	0.123	0.132	0.117	0.123	0.170	0.115	0.111	0.494	0.117	0.229	0.642	0.159	0.317			
TSP	0.451	0.443	0.601	0.493	0.500	0.667	0.473	0.186	0.529	0.306	0.239	0.613	0.255	0.087		
TU	0.197	0.237	0.361	0.418	0.294	0.364	0.290	0.352	0.317	0.077	0.333	0.186	0.172	0.398	0.220	

**Table 8:** Outer/factor loadings (HOC)—reflective.

	**KJ**	**JK**	**Original**
KA <- KM	0.887	0.886	0.882
KAP <- KM	0.866	0.867	0.875
KD <- KM	0.861	0.861	0.857
LF <- inhibitors	0.854	0.854	0.854
TIF <- inhibitors	0.789	0.789	0.789
TSP <- inhibitors	0.807	0.807	0.807
RO <- istressors	0.761	0.762	0.763
TC <- istressors	0.901	0.901	0.900
TIN <- istressors	0.771	0.771	0.770

**Table 9:** Cronbach alpha, composite reliability, and average variance extracted (HOC).

	**Cronbach alpha**			**Composite reliability**			**Average variance extracted**		
	**KJ**	**JK**	**Original**	**KJ**	**JK**	**Original**	**KJ**	**JK**	**Original**
Inhibitors	0.752	0.752	0.752	0.858	0.858	0.858	0.668	0.668	0.668
KM	0.841	0.842	0.842	0.904	0.905	0.904	0.759	0.760	0.759
istressors	0.750	0.750	0.750	0.854	0.854	0.854	0.662	0.662	0.662

**Table 10:** HTMT ratio (HOC)—reflective.

	**Inhibitors**	**JS**	**KM**	**istressors**
*KJ*				
Inhibitors				
JS	0.553			
KM	0.864	0.585		
istressors	0.396	0.323	0.362	

*JK*				
Inhibitors				
JS	0.553			
KM	0.864	0.585		
istressors	0.396	0.323	0.362	

*Original*				
Inhibitors				
JS	0.553			
KM	0.864	0.584		
istressors	0.395	0.323	0.363	

**Table 11:** VIF/validation of HOC (formative) IC.

	**VIF**	**Outer weights**	**T** ** statistics**	*p * ** values**	**Outer loading**	*p * ** values**
*KJ*						
HC -> IC	2.443	0.403	4.921	~0.001	0.906	~0.001
RC -> IC	2.083	0.190	2.485	0.006	0.806	~0.001
SC -> IC	2.555	0.513	5.767	~0.001	0.939	~0.001

*JK*						
HC -> IC	2.442	0.402	4.917	~0.001	0.906	~0.001
RC -> IC	2.083	0.190	2.485	0.006	0.806	~0.001
SC -> IC	2.555	0.514	5.776	~0.001	0.939	~0.001

*Original*						
HC -> IC	2.442	0.402	4.984	~0.001	0.906	~0.001
RC -> IC	2.083	0.188	2.477	0.007	0.805	~0.001
SC -> IC	2.555	0.516	5.895	~0.001	0.940	~0.001

**Table 12:** Psychometric scale for MISS.

	**istressors**		**SD**	**D**	**N**	**A**	**SA**
	**Meaning**	**Questions**					
TC1	**Technocomplexity:** Depicts situations where IT forces IT professionals/users to spend more time and effort in learning and understanding how to use new applications and technologies.	I do not know enough about this technology to handle my job satisfactorily	(1)	(2)	(3)	(4)	(5)
TC2		I need a long time to understand and use new technologies	(1)	(2)	(3)	(4)	(5)
TC3		I do not find enough time to study and upgrade my technology skills	(1)	(2)	(3)	(4)	(5)
TC5		I often find it too complex to understand and use new technologies	(1)	(2)	(3)	(4)	(5)

TIN1	**Technoinsecurity:** Depicts the complex or phobia IT users/professionals have with the use of new technologies.	I feel constant threat to my job due to new technologies	(1)	(2)	(3)	(4)	(5)
TIN3		I am threatened by coworkers/peers with newer technology skills	(1)	(2)	(3)	(4)	(5)
TIN4		I do not share my knowledge with my coworkers/peers for fear of being replaced	(1)	(2)	(3)	(4)	(5)
TIN5		I feel there is less sharing of knowledge among coworkers/peers for fear of being replaced	(1)	(2)	(3)	(4)	(5)

RO2	**Role overload:** Depicts situations where IT enables users/professionals to perform multiple roles simultaneously.	I have to do things that I do not have enough time and energy for	(1)	(2)	(3)	(4)	(5)
RO3		I need more hours in the day to do all the things that are expected of me	(1)	(2)	(3)	(4)	(5)
RO4		I never seem to catch up	(1)	(2)	(3)	(4)	(5)

*Stress inhibitors*							
LF1	**Literacy facilitation:** Mechanisms provided by organization management to help IT users or professionals cope with the demands of learning about new technology.	Our organization encourages knowledge sharing to help deal with new technology	(1)	(2)	(3)	(4)	(5)
LF2		Our organization emphasizes teamwork in dealing with new technology-related problems	(1)	(2)	(3)	(4)	(5)
LF3		Our organization provides end-user training before the introduction of new technology	(1)	(2)	(3)	(4)	(5)
LF4		Our organization foster a good relationship between IT department and end-users	(1)	(2)	(3)	(4)	(5)
LF5		Our organization provides clear documentation to end-user on using new technologies	(1)	(2)	(3)	(4)	(5)

TSP1	**Technical support provision:** Mechanisms provided by organization management to address users anxiety about potentially disruptive mistakes and technical problems	Our end-user help desk does a good job of answering questions about technology	(1)	(2)	(3)	(4)	(5)
TSP2		Our end-user help desk is well staffed by knowledgeable individuals	(1)	(2)	(3)	(4)	(5)
TSP3		Our end-user help desk is easily accessible	(1)	(2)	(3)	(4)	(5)
TSP4		Our end-user help desk is responsive to end-user requests	(1)	(2)	(3)	(4)	(5)

TIF1	**Technology involvement facilitation:** Mechanisms provided by organization management to keep users informed and familiar with new technology	Our end-users are encouraged to try out new technologies	(1)	(2)	(3)	(4)	(5)
TIF2		Our end-users are rewarded for using new technologies	(1)	(2)	(3)	(4)	(5)
TIF3		Our end-users are consulted before introduction of new technology	(1)	(2)	(3)	(4)	(5)
TIF4		Our end-users are involved in technology change and/or implementation	(1)	(2)	(3)	(4)	(5)

*Job satisfaction*							
JS2	**Job satisfaction:** Describes how pleased a worker is with his or her position of employment in the organization or an evaluation of perceived job characteristics, work environment, and emotional experiences	I like doing the things I do while working	(1)	(2)	(3)	(4)	(5)
JS3		I feel a sense of pride in doing my job	(1)	(2)	(3)	(4)	(5)
JS4		My job is enjoyable	(1)	(2)	(3)	(4)	(5)
JS5		I am satisfied with the feeling of accomplishment I get from my job	(1)	(2)	(3)	(4)	(5)

*Knowledge management behavior*							
KA3	**Knowledge acquisition** Knowledge acquisition consists of managing and using existing information and capturing new ones.	The organization encourages the employees to join formal or informal networking made up by professionals and experience users from outside the organization	(1)	(2)	(3)	(4)	(5)
KA6		There is a consolidated and resourceful Research & Development (R & D) policy	(1)	(2)	(3)	(4)	(5)
KA7		New ideas and approaches on work performance are experienced continuously	(1)	(2)	(3)	(4)	(5)
KA8		The organizational systems and procedures support innovation	(1)	(2)	(3)	(4)	(5)

KD1	**Knowledge Distribution** Knowledge distribution refers to managing the sharing of information in an organization, to prompt innovative and creative ideas.	All employees are informed about the aims of the company	(1)	(2)	(3)	(4)	(5)
KD2		Meetings are periodically held to inform all the employees about the latest innovations in the company	(1)	(2)	(3)	(4)	(5)
KD3		The company has formal mechanisms to guarantee the sharing of the best practices among the different fields of the activity	(1)	(2)	(3)	(4)	(5)
KD4		Information technology is used to improve the flow of information and to encourage communication between individuals within the company	(1)	(2)	(3)	(4)	(5)

KAP1	**Knowledge Application** It entails the integration of the knowledge obtained from both the acquisition and distribution phases into daily business processes to enhance the firm's efficiency and effectiveness.	Our organization always apply the latest technology in the market/or our organization is always up-to-date in technology application	(1)	(2)	(3)	(4)	(5)
KAP2		Our employees are well trained in the latest knowledge in their respective position for better job performance	(1)	(2)	(3)	(4)	(5)
KAP3		Our training process is relevant and effective to improve performance and productivity	(1)	(2)	(3)	(4)	(5)
KAP4		Our organization has processes for applying experimental knowledge	(1)	(2)	(3)	(4)	(5)
KAP5		Our organization has processes for applying knowledge to solve new problems	(1)	(2)	(3)	(4)	(5)

*Intellectual capital*							
HC6	**Human capital:** The summation of employees' skill, capabilities, experience, education, and attitude towards life and business.	Our employees are motivated to share new ideas	(1)	(2)	(3)	(4)	(5)
HC7		Our employees have innovative ideas	(1)	(2)	(3)	(4)	(5)
HC8		Managers in our organization make sure that employees are happy	(1)	(2)	(3)	(4)	(5)
HC9		Managers in our organization understands all factors of employee satisfaction	(1)	(2)	(3)	(4)	(5)
HC11		Our employees are generally happy to work	(1)	(2)	(3)	(4)	(5)
HC12		Our employees are happy to put extra effort when needed	(1)	(2)	(3)	(4)	(5)

RC2	**Relational capital:** The output of the organization's relationship with customers, partners, shareholders, and other stakeholders that are critical to the organizational performance	Our organization continuously meet customers	(1)	(2)	(3)	(4)	(5)
RC3		Our organization places a great focus on customers' feedback	(1)	(2)	(3)	(4)	(5)
RC4		Customers' feedback is shared across departments in our organization	(1)	(2)	(3)	(4)	(5)
RC5		Employees enhance their capabilities through interaction	(1)	(2)	(3)	(4)	(5)
RC6		Employees solve problems through cooperation	(1)	(2)	(3)	(4)	(5)
RC7		Our organization's total customer base is improving	(1)	(2)	(3)	(4)	(5)

SC4	**Structural capital:** The mechanism put in place by the organization that helps to support employees for optimum intellectual performance	Our organization continuously develops new products and services	(1)	(2)	(3)	(4)	(5)
SC5		Our organization support innovative ideas	(1)	(2)	(3)	(4)	(5)
SC6		Our organization constantly improves service quality	(1)	(2)	(3)	(4)	(5)
SC7		Our organization embeds much of its information in structures and systems	(1)	(2)	(3)	(4)	(5)
SC9		Our organization possess processes to develop its unique capabilities	(1)	(2)	(3)	(4)	(5)
SC10		Our organization culture and atmosphere are supportive and comfortable	(1)	(2)	(3)	(4)	(5)
SC12		Our organization is embedded with the latest information technology software	(1)	(2)	(3)	(4)	(5)
SC14		Our organizational systems and procedures support innovation	(1)	(2)	(3)	(4)	(5)
SC15		Employees of our organization are highly empowered	(1)	(2)	(3)	(4)	(5)
SC16		Employees of our organization are stimulated to take initiatives	(1)	(2)	(3)	(4)	(5)

**Table 13:** The forms of the CMISS model, associated hypothesis, and their VIF values.

**Barest form**		**JS (Hypothesis** 1 **)**		**KM (Hypothesis** 2 **)**	
	**VIF**		**VIF**		**VIF**
istressors -> IC	1.000	Inhibitors -> IC	1.307	Inhibitors -> IC	1.973
		JS -> IC	1.295	KM -> IC	1.972
*Barest form with moderator*		istressors -> IC	1.153	Istressors -> IC	1.146
Inhibitors -> IC	1.103	istressors -> JS	1.000	istressors -> KM	1.000
istressors -> IC	1.125	Inhibitors x istressors -> IC	1.160	Inhibitors x istressors -> IC	1.283
Inhibitors x istressors -> IC	1.022	Inhibitors x JS -> IC	1.157	Inhibitors x KM -> IC	1.265

**JS||KM (Hypothesis** 3 **)**		**KM-JS (Hypothesis** 4 **)**		**JS-KM (Hypothesis** 5 **)**	
	**VIF**		**VIF**		**VIF**
Inhibitors -> IC	2.021	Inhibitors -> IC	2.023	Inhibitors -> IC	2.023
JS -> IC	1.391	JS -> IC	1.396	JS -> IC	1.395
KM -> IC	2.122	KM -> IC	2.138	JS -> KM	1.079
istressors -> IC	1.163	KM -> JS	1.105	KM -> IC	2.136
istressors -> JS	1.000	istressors -> IC	1.163	istressors -> IC	1.163
istressors -> KM	1.000	istressors -> JS	1.105	istressors -> JS	1.000
Inhibitors x istressors -> IC	1.291	istressors -> KM	1.000	istressors -> KM	1.079
Inhibitors x JS -> IC	1.718	Inhibitors x istressors -> IC	1.291	Inhibitors x istressors -> IC	1.292
Inhibitors x KM -> IC	1.864	Inhibitors x JS -> IC	1.737	Inhibitors x JS -> IC	1.735
		Inhibitors x KM -> IC	1.882	Inhibitors x KM -> IC	1.880

**Table 14:** TTS path, total effects, and explanatory power coefficients.

**Barest form**				
*Direct path*				
	*Β*	SE	*T* statistics	*p* values
istressors -> IC	−0.323	0.070	4.584	~0.001

*Total effect*				
istressors -> IC	−0.323	0.070	4.584	~0.001

*Model explanatory power*				
Predictor(s)	Outcome(s)	*R* ^2^	*f* ^2^	*Q* ^2^
istressors	IC	0.104	0.116	0.067

**Table 15:** TTSC paths, total effects, and explanatory power coefficients.

**Barest form with moderator**				
*Direct path*				
	*β*	SE	*T* statistics	*p* values
Inhibitor -> IC	0.645	0.046	14.155	~0.001
istressors -> IC	−0.125	0.069	1.810	0.035
Inhibitors x istressors -> IC	0.016	0.059	0.269	0.394

*Total effect*				
Inhibitor -> IC	0.645	0.046	14.155	~0.001
istressors -> IC	−0.125	0.069	1.810	0.035
Inhibitors x istressors -> IC	0.016	0.059	0.269	0.394

*Model explanatory power*				
Predictor(s)	Outcome(s)	*R* ^2^	*f* ^2^	*Q* ^2^
istressors	IC	0.479	0.026	0.439
Inhibitors			0.723	
Inhibitors x istressors			0.000	

**Table 16:** Job satisfaction–mediated TTSC paths, total effects, and explanatory power coefficients.

**JS mediation only**				
*Direct path*				
	*β*	SE	*T* statistics	*p* values
Inhibitors -> IC	0.582	0.057	10.260	~0.001
JS -> IC	0.158	0.073	2.158	0.015
istressors -> IC	−0.101	0.065	1.549	0.061
istressors -> JS	−0.271	0.062	4.352	~0.001
Inhibitors x JS -> IC	0.050	0.045	1.110	0.133
Inhibitors x istressors -> IC	0.044	0.061	0.720	0.236

*Indirect path*				
istressor ->JS ->IC	−0.043	0.022	1.923	0.027

*Total effect*				
Inhibitors -> IC	0.582	0.057	10.260	~0.001
JS -> IC	0.158	0.073	2.158	0.015
istressors -> IC	−0.144	0.071	2.036	0.021
istressors -> JS	−0.271	0.062	4.352	~0.001
Inhibitors x JS -> IC	0.050	0.045	1.110	0.133
Inhibitors x istressors -> IC	0.044	0.061	0.720	0.236

*Model explanatory power*				
Predictor(s)	Outcome(s)	*R* ^2^	*f* ^2^	*Q* ^2^
JS	IC	0.501	0.039	0.438
Inhibitor			0.520	
istressors			0.018	
Inhibitor x JS			0.005	
Inhibitor x istressors			0.003	
istressors	JS	0.074	0.079	0.059

**Table 17:** Knowledge management–mediated TTSC paths, total effects, and explanatory power coefficients.

**KM mediation only**				
*Direct path*				
	*β*	SE	*T* statistics	*p* values
Inhibitors -> IC	0.223	0.066	3.367	~0.001
KM -> IC	0.648	0.061	10.585	~0.001
istressors -> IC	−0.056	0.053	1.060	0.145
istressors -> KM	−0.309	0.070	4.448	~0.001
Inhibitors x KM -> IC	0.118	0.039	3.022	0.001
Inhibitors x istressors -> IC	0.095	0.053	1.774	0.038

*Indirect path*				
istressor ->KM ->IC	−0.200	0.048	4.136	~0.001

*Total effect*				
Inhibitors -> IC	0.223	0.066	3.367	~0.001
KM -> IC	0.648	0.061	10.585	~0.001
istressors -> IC	−0.256	0.069	3.733	~0.001
istressors -> KM	−0.309	0.070	4.448	~0.001
Inhibitors x KM -> IC	0.118	0.039	3.022	0.001
Inhibitors x istressors -> IC	0.095	0.053	1.774	0.038

*Model explanatory power*				
Predictor(s)	Outcome(s)	*R* ^2^	*f* ^2^	*Q* ^2^
KM	IC	0.706	0.723	0.293
Inhibitor			0.086	
istressors			0.009	
Inhibitor x KM			0.044	
Inhibitor x istressors			0.022	
istressors	KM	0.096	0.106	0.081

**Table 18:** Knowledge management and job satisfaction in parallel mediation of TTSC paths, total effects, and explanatory power coefficients.

**JS and KM parallel mediation**				
*Direct path*				
	*β*	SE	*T* statistics	*p* values
Inhibitors -> IC	0.217	0.067	3.240	0.001
JS -> IC	0.020	0.067	0.295	0.384
KM -> IC	0.644	0.069	9.397	~0.001
istressors -> IC	−0.057	0.051	1.119	0.132
istressors -> JS	−0.272	0.063	4.337	~0.001
istressors -> KM	−0.309	0.071	4.338	~0.001
Inhibitors x istressors -> IC	0.090	0.053	1.705	0.044
Inhibitors x JS -> IC	−0.043	0.052	0.828	0.204
Inhibitors x KM -> IC	0.142	0.056	2.534	0.006

*Indirect path*				
Istressor ->KM ->IC	−0.199	0.050	3.992	~0.001
Istressor ->JS ->IC	−0.005	0.019	0.279	0.390

*Total effect*				
Inhibitors -> IC	0.217	0.067	3.240	0.001
JS -> IC	0.020	0.067	0.295	0.384
KM -> IC	0.644	0.069	9.397	~0.001
istressors -> IC	−0.261	0.070	3.722	~0.001
istressors -> JS	−0.272	0.063	4.337	~0.001
istressors -> KM	−0.309	0.071	4.338	~0.001
Inhibitors x istressors -> IC	0.090	0.053	1.705	0.044
Inhibitors x JS -> IC	−0.043	0.052	0.828	0.204
Inhibitors x KM -> IC	0.142	0.056	2.534	0.006

*Model explanatory power*				
Predictor(s)	Outcome(s)	*R* ^2^	*f* ^2^	*Q* ^2^
KM	IC	0.707	0.667	0.289
JS			0.001	
Inhibitor			0.080	
istressors			0.010	
Inhibitor x KM			0.044	
Inhibitor x JS			0.004	
Inhibitor x istressors			0.020	
istressors	KM	0.095	0.105	0.081
istressors	JS	0.074	0.080	0.060

**Table 19:** TTSC knowledge management mediating job satisfaction–mediated TTSC paths, total effects, and explanatory power coefficients.

**KM-JS–mediated mediation**				
*Direct path*				
	*β*	SE	*T* statistics	*p* values
Inhibitors -> IC	0.219	0.056	3.281	0.001
JS -> IC	0.017	0.068	0.250	0.401
KM -> IC	0.642	0.068	9.412	~0.001
KM -> JS	0.453	0.067	6.753	~0.001
istressors -> IC	−0.058	0.051	1.132	0.129
istressors -> JS	−0.131	0.063	2.065	0.019
istressors -> KM	−0.308	0.071	4.343	~0.001
Inhibitors x istressors -> IC	0.090	0.053	1.699	0.045
Inhibitors x JS -> IC	−0.045	0.053	0.854	0.197
Inhibitors x KM -> IC	0.144	0.056	2.569	0.005

*Indirect path*				
KM ->JS ->IC	0.008	0.032	0.244	0.404
istressor ->KM ->IC	−0.198	0.050	3.990	~0.001
istressor ->JS ->IC	−0.002	0.010	0.223	0.412
istressor ->KM -> JS -> IC	−0.002	0.010	0.231	0.409
istressor ->KM ->JS	−0.140	0.040	3.494	~0.001

*Total effect*				
Inhibitors -> IC	0.219	0.067	3.281	0.001
JS -> IC	0.017	0.068	0.250	0.401
KM -> IC	0.650	0.061	10.717	~0.001
KM -> JS	0.453	0.067	6.753	~0.001
istressors -> IC	−0.261	0.070	3.719	~0.001
istressors -> JS	−0.271	0.063	4.280	~0.001
istressors -> KM	−0.308	0.071	4.343	~0.001
Inhibitors x istressors -> IC	0.090	0.053	1.699	0.045
Inhibitors x JS -> IC	−0.045	0.053	0.854	0.197
Inhibitors x KM -> IC	0.144	0.056	2.569	0.005

*Model explanatory power*				
Predictor(s)	Outcome(s)	*R* ^2^	*f* ^2^	*Q* ^2^
KM	IC	0.705	0.654	0.290
JS			0.001	
Inhibitor			0.080	
istressors			0.010	
Inhibitor x KM			0.044	
Inhibitor x JS			0.004	
Inhibitor x istressors			0.020	
istressors	JS	0.095	0.021	0.081
KM			0.251	
istressors	KM	0.259	0.105	0.061

**Table 20:** TTSC job satisfaction mediating knowledge management–mediated TTSC paths, total effects, and explanatory power coefficients.

**JS-KM–mediated mediation**				
*Direct path*				
	*β*	SE	*T* statistics	*p* values
Inhibitors -> IC	0.218	0.067	3.273	0.001
JS -> IC	0.017	0.068	0.254	0.400
JS -> KM	0.442	0.067	6.553	~0.001
KM -> IC	0.643	0.068	9.423	~0.001
istressors -> IC	−0.058	0.051	1.132	0.129
istressors -> JS	−0.271	0.063	4.306	~0.001
istressors -> KM	−0.189	0.072	2.632	0.004
Inhibitors x JS -> IC	−0.045	0.053	0.857	0.196
Inhibitors x KM -> IC	0.144	0.056	2.574	0.005
Inhibitors x istressors -> IC	0.090	0.053	1.698	0.045

*Indirect path*				
Istressor ->KM ->IC	−0.121	0.046	2.620	0.004
Istressor ->JS ->IC	−0.005	0.019	0.241	0.405
Istressor ->JS -> KM -> IC	−0.077	0.026	3.003	0.001
Istressor ->JS ->KM	−0.120	0.035	3.416	~0.001
JS ->KM ->IC	0.284	0.055	5.140	~0.001

*Total effect*				
Inhibitors -> IC	0.218	0.067	3.273	0.001
JS -> IC	0.301	0.069	4.366	~0.001
JS -> KM	0.442	0.067	6.553	~0.001
KM -> IC	0.643	0.068	9.423	~0.001
istressors -> IC	-0.261	0.070	3.720	~0.001
istressors -> JS	-0.271	0.063	4.306	~0.001
istressors -> KM	-0.308	0.071	4.341	~0.001
Inhibitors x JS -> IC	-0.045	0.053	0.857	0.196
Inhibitors x KM -> IC	0.144	0.056	2.574	0.005
Inhibitors x istressors -> IC	0.090	0.053	1.698	0.045

*Model explanatory power*				
Predictor(s)	Outcome(s)	*R* ^2^	*f* ^2^	*Q* ^2^
KM	IC	0.705	0.656	0.290
JS			0.001	
Inhibitor			0.080	
istressors			0.010	
Inhibitor x KM			0.045	
Inhibitor x JS			0.004	
Inhibitor x istressors			0.020	
istressors	KM	0.276	0.046	0.081
JS			0.249	
istressors	JS	0.073	0.079	0.061

## Data Availability

The data that support the findings of this study are available from the corresponding author upon reasonable request.
