# Supplementary material for: Psychometric Model for Service Firm's Intellectual Stress Diagnosis and Management: Development and Validation
Source: ScientificWorldJournal. 2025 May 24;2025:1117495. doi: 10.1155/tswj/1117495 (PMC12126267; doi:10.1155/tswj/1117495)
Supplement: Supporting Information — Additional supporting information can be found online in the Supporting Information section. Questionnaire with demographics and Likert. [file 1117495.f1.docx]

Appendix B: Questionnaire with demographics and Likert

**SURVEY QUESTIONNAIRE**

My name is EMUOBONUVIE Erhinyoja Andy and I am a PhD student in the Department of Computer Science, Faculty of Physical Sciences, University of Benin (UNIBEN), Benin City, Edo State, Nigeria.

As part of my studies towards a PhD in Computer Science at UNIBEN, I am presently conducting a survey for my PhD thesis under the supervision of Professor Godspower O. Ekuobase, PhD. This research is aimed at enabling the effective management of Intellectual stress among professionals/users of Information Technology.

Information and communication technology (ICT) now drives organizational value, particularly of service firms. However, the continuous influx and exhilarating operational changes of these technologies forces serious strain on the physical and intellectual capability of its users and professionals. This questionnaire is intended to elicit information from Information Technology (IT) users/professionals with a view to effectively manage this disservice of ICT.

If you have any questions or concerns, please contact me at [andy.emuobonuvie@unidel.edu.ng](mailto:andy.emuobonuvie@unidel.edu.ng)

**Kindly be our respondent**

**Instructions**:

1) This questionnaire consists of two sections: section A and section B.

2) It is estimated that this questionnaire will take approximately 15 to 20 minutes to complete.

3) The contents of this questionnaire will be kept **strictly confidential** for this investigation.

**Section A: Demographic Properties/Organization Information**

This section is seeking to elicit some of your demographic properties and that of your organization.

Q1. **Gender:** Female Male Others (Specify)__________

Q2. **Age:** Below 20 years old 20–29 years old 30–39 years old

40 – 49 years old 50 – 59 years old 60 years and above

Q3. **Highest education qualification:**

High/Secondary school Diploma/Advance diploma

Bachelor’s degree Master’s degree

PhD degree

Q4. **What is the name of the organization on whose behalf you are answering these questions?**

Please specify _______________________________________________________

Q5. **What is the type of organization? (e.g consultancy services, Telecommunications)**

Please specify ______________________________________________________

Q6. **Please specify the level of Management being assessed**

| Operational Level |  |
| --- | --- |
| Middle Management |  |
| Senior Management |  |

Q7. Are you an IT Staff? (An IT Staff is any person whose job function is almost solely dependent on Information Technology

Yes No

**SECTION B**

| **Item Number** | Dear Respondent,  Please indicate your level of agreement or disagreement with the questions below by checking [√] the appropriate option according to the scoring given below.  **[(1) = Strongly Disagree, (2) = Disagree, (3) = Neutral/Undecided,**  **(4) = Agree, (5) = Strongly Agree]** | | **Strongly Disagree** | **Disagree** | **Neutral/Undecided** | **Agree** | **Strongly Agree** |
| --- | --- | --- | --- | --- | --- | --- | --- |
| **Stressors** | | | | | | | |
|  | **Meaning** | **Questions** |  |  |  |  |  |
| TO1 | **Techno-Overload:**  Depicts situation where IT users/professionals are exposed to more IT that can make them work more and faster than they can conveniently handle | I am forced by this technology to work much faster | (1) | (2) | (3) | (4) | (5) |
| TO2 |  | I am forced by this technology to do more work than I can handle | (1) | (2) | (3) | (4) | (5) |
| TO3 |  | I am forced by this technology to work with very tight time schedule | (1) | (2) | (3) | (4) | (5) |
| TO4 |  | I am forced to change my work habits to adapt to new technologies | (1) | (2) | (3) | (4) | (5) |
| TO5 |  | I have a higher workload because of increased technology. | (1) | (2) | (3) | (4) | (5) |
| TI1 | **Techno-Invasion**  Depicts situations where IT users/professionals can work or be reached anywhere and anytime and feel the need to be constantly connected. | I spend less time with my family due to use of technology | (1) | (2) | (3) | (4) | (5) |
| TI2 |  | I have to be in touch with my work even during vacations due to the use of technology | (1) | (2) | (3) | (4) | (5) |
| TI3 |  | I have to sacrifice my vacation and weekend time to keep current on new technologies. | (1) | (2) | (3) | (4) | (5) |
| TI4 |  | I feel my personal life is being invaded by use of technology | (1) | (2) | (3) | (4) | (5) |
| TC1 | **Techno-Complexity**  Depicts situations where IT forces IT professionals/users to spend more time and effort in learning and understanding how to use new applications and technologies. | I do not know enough about this technology to handle my job satisfactorily | (1) | (2) | (3) | (4) | (5) |
| TC2 |  | I need a long time to understand and use new technologies | (1) | (2) | (3) | (4) | (5) |
| TC3 |  | I do not find enough time to study and upgrade my technology skills | (1) | (2) | (3) | (4) | (5) |
| TC4 |  | I find new recruits to this organization know more about the technology than I do | (1) | (2) | (3) | (4) | (5) |
| TC5 |  | I often find it too complex to understand and use new technologies | (1) | (2) | (3) | (4) | (5) |
| TIN1 | **Techno-Insecurity**  Depicts the complex or phobia IT users/professionals have with the use of new technologies. | I feel constant threat to my job due to new technologies | (1) | (2) | (3) | (4) | (5) |
| TIN2 |  | I have to constantly update my skills to avoid being replaced | (1) | (2) | (3) | (4) | (5) |
| TIN3 |  | I am threatened by coworkers/peers with newer technology skills | (1) | (2) | (3) | (4) | (5) |
| TIN4 |  | I do not share my knowledge with my coworkers/peers for fear of being replaced | (1) | (2) | (3) | (4) | (5) |
| TIN5 |  | I feel there is less sharing of knowledge among coworkers/peers for fear of being replaced | (1) | (2) | (3) | (4) | (5) |
| TU1 | **Techno-Uncertainty**  Depicts situations where the IT skillset required by IT users/professionals is constantly changing and rapidly so. | There are always new entrance/development in the technologies used in our organization | (1) | (2) | (3) | (4) | (5) |
| TU2 |  | There are constant changes in computer software in our organization | (1) | (2) | (3) | (4) | (5) |
| TU3 |  | There are constant changes in computer hardware in our organization | (1) | (2) | (3) | (4) | (5) |
| TU4 |  | There is frequent upgrades in computer network in our organization | (1) | (2) | (3) | (4) | (5) |
| RO1 | **Role-Overload**  Depicts situations where IT enables users/professionals to perform multiple roles simultaneously. | I perform multiple roles with the help of technology | (1) | (2) | (3) | (4) | (5) |
| RO2 |  | I have to do things that I do not have enough time and energy for. | (1) | (2) | (3) | (4) | (5) |
| RO3 |  | I need more hours in the day to do all the things that are expected of me. | (1) | (2) | (3) | (4) | (5) |
| RO4 |  | I never seem to catch up. | (1) | (2) | (3) | (4) | (5) |
| RO5 |  | There are times when I cannot meet everyone’s expectations | (1) | (2) | (3) | (4) | (5) |
| **Stress Inhibitors** | | | | | | | |
| LF1 | **Literacy Facilitation:**  Mechanisms provided by organization management to help IT users or professionals cope with the demands of learning about new technology. | Our organization encourages knowledge sharing to help deal with new technology | (1) | (2) | (3) | (4) | (5) |
| LF2 |  | Our organization emphasizes teamwork in dealing with new technology-related problems | (1) | (2) | (3) | (4) | (5) |
| LF3 |  | Our organization provides end-user training before the introduction of new technology. | (1) | (2) | (3) | (4) | (5) |
| LF4 |  | Our organization foster a good relationship between IT department and end users | (1) | (2) | (3) | (4) | (5) |
| LF5 |  | Our organization provides clear documentation to end user on using new technologies. | (1) | (2) | (3) | (4) | (5) |
| TSP1 | **Technical Support Provision.**  Mechanisms provided by organization management to address users anxiety about potentially disruptive mistakes and technical problems | Our end-user help desk does a good job of answering questions about technology | (1) | (2) | (3) | (4) | (5) |
| TSP2 |  | Our end-user help desk is well staffed by knowledgeable individuals | (1) | (2) | (3) | (4) | (5) |
| TSP3 |  | Our end-user help desk is easily accessible | (1) | (2) | (3) | (4) | (5) |
| TSP4 |  | Our end-user help desk is responsive to end-user requests | (1) | (2) | (3) | (4) | (5) |
| TIF1 | **Technology Involvement Facilitation:**  Mechanisms provided by organization management to keep users informed and familiar with new technology | Our end users are encouraged to try out new technologies | (1) | (2) | (3) | (4) | (5) |
| TIF2 |  | Our end users are rewarded for using new technologies | (1) | (2) | (3) | (4) | (5) |
| TIF3 |  | Our end users are consulted before introduction of new technology | (1) | (2) | (3) | (4) | (5) |
| TIF4 |  | Our end users are involved in technology change and/or implementation | (1) | (2) | (3) | (4) | (5) |
| **Job Satisfaction** | | | | | | | |
| JS1 | **Job Satisfaction:**  Describes how pleased a worker is with his or her position of employment in the organization or an evaluation of perceived job characteristics, work environment, and emotional experiences | I always exceed my performance expectation at work place | (1) | (2) | (3) | (4) | (5) |
| JS2 |  | I like doing the things I do while working | (1) | (2) | (3) | (4) | (5) |
| JS3 |  | I feel a sense of pride in doing my job | (1) | (2) | (3) | (4) | (5) |
| JS4 |  | My job is enjoyable | (1) | (2) | (3) | (4) | (5) |
| JS5 |  | I am satisfied with the feeling of accomplishment I get from my job | (1) | (2) | (3) | (4) | (5) |
| **Knowledge Management Behaviour** | | | | | | | |
| KA1 | **Knowledge Acquisition**  Knowledge acquisition consists of managing and using existing information and capturing new ones. | We have a system that allows us to learn successful practices from other organizations. | (1) | (2) | (3) | (4) | (5) |
| KA2 |  | The company is in touch with professionals and expert technicians. | (1) | (2) | (3) | (4) | (5) |
| KA3 |  | The organization encourages the employees to join formal or informal networking made up by professionals and experience users from outside the organization. | (1) | (2) | (3) | (4) | (5) |
| KA4 |  | We often ask our customers what they want or need. | (1) | (2) | (3) | (4) | (5) |
| KA5 |  | The employees attend fairs and exhibitions regularly. | (1) | (2) | (3) | (4) | (5) |
| KA6 |  | There is a consolidated and resourceful Research & Development (R & D) policy. | (1) | (2) | (3) | (4) | (5) |
| KA7 |  | New ideas and approaches on work performance are experienced continuously. | (1) | (2) | (3) | (4) | (5) |
| KA8 |  | The organizational systems and procedures support innovation. | (1) | (2) | (3) | (4) | (5) |
| KD1 | **Knowledge Distribution**  Knowledge distribution refers to managing the sharing of information in an organization, to prompt innovative and creative ideas. | All employees are informed about the aims of the company. | (1) | (2) | (3) | (4) | (5) |
| KD2 |  | Meetings are periodically held to inform all the employees about the latest innovations in the company. | (1) | (2) | (3) | (4) | (5) |
| KD3 |  | The company has formal mechanisms to guarantee the sharing of the best practices among the different fields of the activity. | (1) | (2) | (3) | (4) | (5) |
| KD4 |  | Information technology is used to improve the flow of information and to encourage communication between individuals within the company. | (1) | (2) | (3) | (4) | (5) |
| KD5 |  | There are individuals within the organization who take part in several teams or divisions and act as links between them. | (1) | (2) | (3) | (4) | (5) |
| KD6 |  | There are individuals responsible for collecting, assembling and distributing internal employees’ suggestions. | (1) | (2) | (3) | (4) | (5) |
| KAP1 | **Knowledge Application**  It entails the integration of the knowledge obtained from both the acquisition and distribution phases into daily business processes to enhance the firm’s efficiency and effectiveness. | Our organization always apply the latest technology in the market/or our organization is always up-to-date in technology application. | (1) | (2) | (3) | (4) | (5) |
| KAP2 |  | Our employees are well trained in the latest knowledge in their respective position for better job performance. | (1) | (2) | (3) | (4) | (5) |
| KAP3 |  | Our training process is relevant and effective to improve performance and productivity. | (1) | (2) | (3) | (4) | (5) |
| KAP4 |  | Our organization has processes for applying experimental knowledge. | (1) | (2) | (3) | (4) | (5) |
| KAP5 |  | Our organization has processes for applying knowledge to solve new problems. | (1) | (2) | (3) | (4) | (5) |
| **Intellectual Capital** | | | | | | | |
| HC1 | **Human Capital:**  The summation of employees’ skill, capabilities, experience, education and attitude towards life and business. | Our employees undergo continuous training | (1) | (2) | (3) | (4) | (5) |
| HC2 |  | Our employees are highly educated | (1) | (2) | (3) | (4) | (5) |
| HC3 |  | Our employee skills are always upgraded | (1) | (2) | (3) | (4) | (5) |
| HC4 |  | Our employees are creative and bright | (1) | (2) | (3) | (4) | (5) |
| HC5 |  | Our employees come up with new ideas | (1) | (2) | (3) | (4) | (5) |
| HC6 |  | Our employees are motivated to share new ideas | (1) | (2) | (3) | (4) | (5) |
| HC7 |  | Our employees have innovative ideas | (1) | (2) | (3) | (4) | (5) |
| HC8 |  | Managers in our organization make sure that employees are happy | (1) | (2) | (3) | (4) | (5) |
| HC9 |  | Managers in our Organization understands all factors of employees satisfaction | (1) | (2) | (3) | (4) | (5) |
| HC10 |  | Managers in our Organization help employees in solving official problems | (1) | (2) | (3) | (4) | (5) |
| HC11 |  | Our employees are generally happy to work | (1) | (2) | (3) | (4) | (5) |
| HC12 |  | Our employees are happy to put extra effort when needed | (1) | (2) | (3) | (4) | (5) |
| HC13 |  | Our employees are devoted to their work | (1) | (2) | (3) | (4) | (5) |
| RC1 | **Relational Capital:**  The output of the organization’s relationship with customers, partners, shareholders, and other stakeholders that are critical to the organizational performance | Data about customers are continuously updated in our organization | (1) | (2) | (3) | (4) | (5) |
| RC2 |  | Our organization continuously meet customers | (1) | (2) | (3) | (4) | (5) |
| RC3 |  | Our organization places a great focus on customers’ feedback | (1) | (2) | (3) | (4) | (5) |
| RC4 |  | Customers’ feedback is shared across departments in our organization | (1) | (2) | (3) | (4) | (5) |
| RC5 |  | Employees enhance their capabilities through interaction | (1) | (2) | (3) | (4) | (5) |
| RC6 |  | Employees solve problems through cooperation | (1) | (2) | (3) | (4) | (5) |
| RC7 |  | Our organization's total customer base is improving | (1) | (2) | (3) | (4) | (5) |
| SC1 | **Structural Capital:**  The mechanism put in place by the organization that helps to support employees for optimum intellectual performance | The atmosphere in our organization is pleasant | (1) | (2) | (3) | (4) | (5) |
| SC2 |  | In our organization, managers and staff communicate well | (1) | (2) | (3) | (4) | (5) |
| SC3 |  | Knowledge increase is well supported in our organization | (1) | (2) | (3) | (4) | (5) |
| SC4 |  | Our organization continuously develops new products and services | (1) | (2) | (3) | (4) | (5) |
| SC5 |  | Our organization support innovative ideas | (1) | (2) | (3) | (4) | (5) |
| SC6 |  | Our organization constantly improves service quality | (1) | (2) | (3) | (4) | (5) |
| SC7 |  | Our organization embeds much of its information in structures and systems | (1) | (2) | (3) | (4) | (5) |
| SC8 |  | Employees have access to the information system whenever needed | (1) | (2) | (3) | (4) | (5) |
| SC9 |  | Our organization possess processes to develop its unique capabilities | (1) | (2) | (3) | (4) | (5) |
| SC10 |  | Our organization culture and atmosphere are supportive and comfortable | (1) | (2) | (3) | (4) | (5) |
| SC11 |  | Our organization uses computers for operational purposes | (1) | (2) | (3) | (4) | (5) |
| SC12 |  | Our organization is embedded with the latest information technology software. | (1) | (2) | (3) | (4) | (5) |
| SC13 |  | Information Technology contributes to service quality in our organization | (1) | (2) | (3) | (4) | (5) |
| SC14 |  | Our organizational systems and procedures support innovation | (1) | (2) | (3) | (4) | (5) |
| SC15 |  | Employees of our organization are highly empowered | (1) | (2) | (3) | (4) | (5) |
| SC16 |  | Employees of our organization are stimulated to take initiatives | (1) | (2) | (3) | (4) | (5) |
